# Distinct regions of the cerebellum show gray matter decreases in autism, ADHD, and developmental dyslexia

**DOI:** 10.3389/fnsys.2014.00092

**Published:** 2014-05-20

**Authors:** Catherine J. Stoodley

**Affiliations:** Department of Psychology, American UniversityWashington, DC, USA

**Keywords:** cerebellum, autism spectrum disorder, attention deficit hyperactivity disorder, developmental dyslexia, meta-analysis

## Abstract

Differences in cerebellar structure have been identified in autism spectrum disorder (ASD), attention deficit hyperactivity disorder (ADHD), and developmental dyslexia. However, it is not clear if different cerebellar regions are involved in each disorder, and thus whether cerebellar anatomical differences reflect a generic developmental vulnerability or disorder-specific characteristics. To clarify this, we conducted an anatomic likelihood estimate (ALE) meta-analysis on voxel-based morphometry (VBM) studies which compared ASD (17 studies), ADHD (10 studies), and dyslexic (10 studies) participants with age-matched typically-developing (TD) controls. A second ALE analysis included studies in which the cerebellum was a region of interest (ROI). There were no regions of significantly increased gray matter (GM) in the cerebellum in ASD, ADHD, or dyslexia. Data from ASD studies revealed reduced GM in the inferior cerebellar vermis (lobule IX), left lobule VIIIB, and right Crus I. In ADHD, significantly decreased GM was found bilaterally in lobule IX, whereas participants with developmental dyslexia showed GM decreases in left lobule VI. There was no overlap between the cerebellar clusters identified in each disorder. We evaluated the functional significance of the regions revealed in both whole-brain and cerebellar ROI ALE analyses using Buckner and colleagues' 7-network functional connectivity map available in the SUIT cerebellar atlas. The cerebellar regions identified in ASD showed functional connectivity with frontoparietal, default mode, somatomotor, and limbic networks; in ADHD, the clusters were part of dorsal and ventral attention networks; and in dyslexia, the clusters involved ventral attention, frontoparietal, and default mode networks. The results suggest that different cerebellar regions are affected in ASD, ADHD, and dyslexia, and these cerebellar regions participate in functional networks that are consistent with the characteristic symptoms of each disorder.

## Introduction

**Figure 1:**
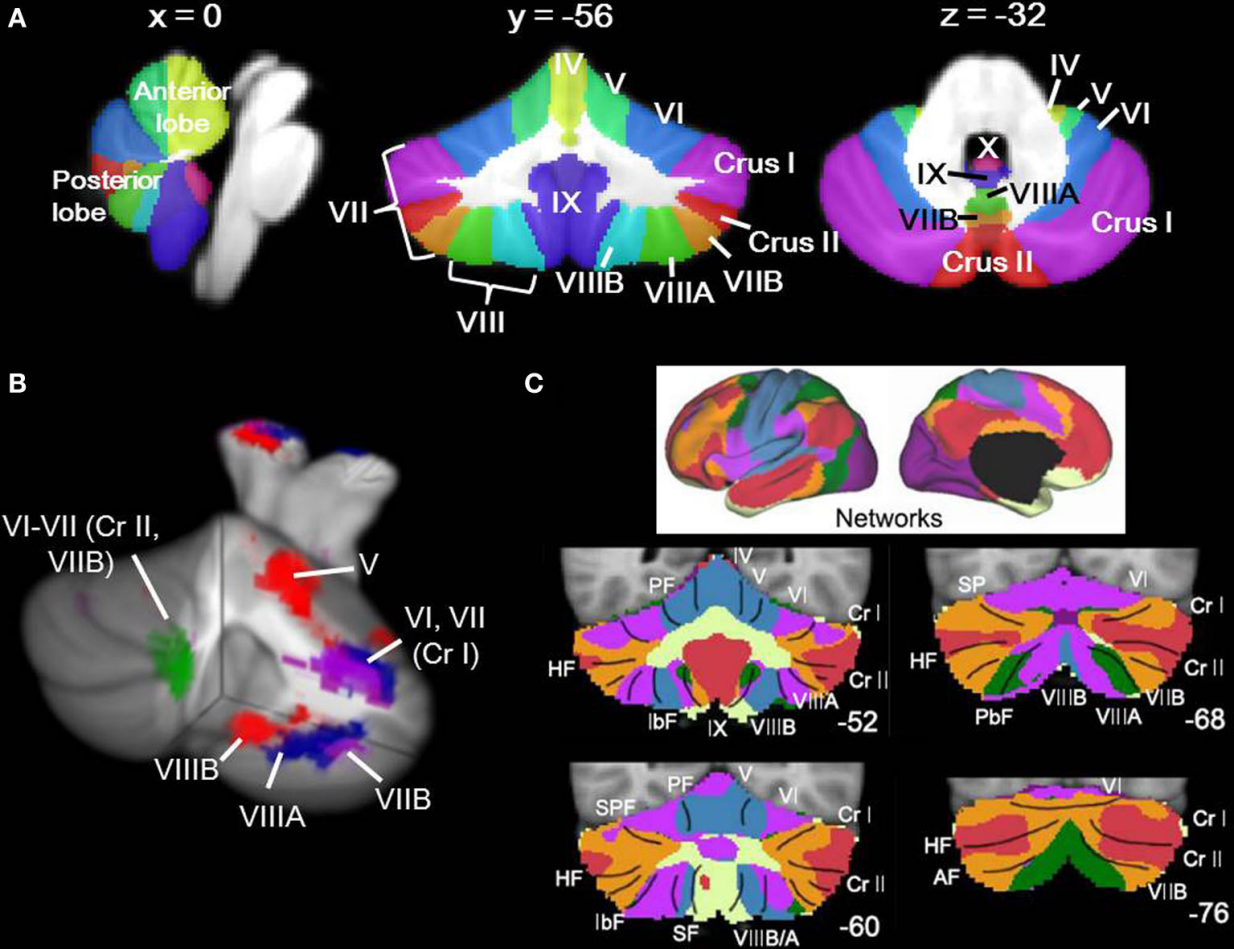
**Cerebellar anatomy and functional topography. (A)** Cerebellar anatomy shown on coronal, sagittal, and axial slices through the Spatially Unbiased Infratentorial (SUIT) atlas (Diedrichsen, 2006; Diedrichsen et al., 2009). The cerebellum is subdivided into three lobes (anterior, posterior, and flocculonodular [lobule X]) and 10 lobules (I-X). In humans, lobule VII is subdivided into Crus I, Crus II, and VIIB, and lobule VIII is divided into VIIIA and VIIIB. Yellow, lobules I–IV; light green, lobule V; blue, lobule VI; purple, lobule VII (Crus I); red, lobule VII (Crus II); orange, lobule VII (VIIB); green, lobule VIIIA; aqua, lobule VIIIB; dark purple, lobule IX; pink-purple, X. **(B)** Functional topography revealed by task-based neuroimaging (Stoodley et al., 2012b). Activation related to right-handed finger tapping (red), language tasks (blue), working memory (violet), and spatial (green) tasks is shown. **(C)** Functional connectivity of the cerebellum based on correlations with cortical networks (adapted with permission from Buckner et al., 2011). Dark purple, visual; blue, somatomotor; green, dorsal attention; violet, ventral attention; cream, limbic; orange, frontoparietal; red, default network.

Our understanding of the human cerebellum has undergone substantial revision in the past 20 years. Traditionally considered a motor structure, anatomical, clinical, and neuroimaging data have converged to suggest that the cerebellum has a role in modulation of cerebro-cerebellar circuits involved in cognition and emotion as well as motor control (for reviews, see Strick et al., 2009; Stoodley and Schmahmann, 2010). The cerebellum forms closed-loop circuits with the majority of the cerebral cortex, with the cerebellar hemispheres projecting to the contralateral cerebral cortex. This closed-loop circuitry, together with the crystalline structure of the cerebellar cortex, suggests that the cerebellum contains repeating modules (Apps and Garwicz, 2005), such that the function of a given region of the cerebellum depends on its inputs and outputs (for review, Ramnani, 2006). Supporting this concept, within the cerebellum different regions are involved in overt motor control vs. cognitive and emotional processing (Stoodley and Schmahmann, 2009). This functional topography of the human cerebellum is based on its anatomical connections with the cerebral cortex and spinal cord (Stoodley and Schmahmann, 2010): briefly, lobules I–V and lobule VIII are predominantly sensorimotor; lobules VI and VII form circuits with frontal and parietal association cortices; lobule IX may participate in multiple cortical networks, including the default mode network; and lobule X comprises the vestibulocerebellum. Resting-state functional connectivity studies also support this topography, and indicate that the regions of the cerebellum showing correlated activity with sensorimotor cortices differ from those that are functionally related to prefrontal and parietal association areas (e.g., Habas et al., 2009; Buckner et al., 2011). Figure 1 shows cerebellar lobular anatomy, and cerebellar functional topography as revealed by task-based neuroimaging (Figure 1B) and resting state functional connectivity (Figure 1C).

Cerebellar functional topography is of importance when considering the role of the cerebellum in developmental disorders. As early as 1990, Levinson suggested that cerebellar-vestibular testing could potentially be used in the diagnosis of learning disabilities (such as developmental dyslexia) and attention deficit hyperactivity disorder (ADHD; Levinson, 1990). Since then, neuroimaging studies have reported cerebellar structural and functional differences in autism spectrum disorder (ASD), ADHD, and developmental dyslexia (hereafter “dyslexia”). However, it is not clear whether the same or different cerebellar regions are affected in these disorders. Understanding the convergence and divergence of cerebellar structural differences in ASD, ADHD and dyslexia can clarify whether the role of the cerebellum in these disorders is specific to particular cerebro-cerebellar circuits or represents a more general characteristic of a developmental disorder.

ASD is characterized by deficits in communication and social interaction, and repetitive and restrictive behaviors and interests (American Psychiatric Association, 2013). Mounting evidence from early postmortem (Bauman and Kemper, 1985; Ritvo et al., 1986; Bailey et al., 1998) and imaging studies (Courchesne et al., 1988) and more recent genetic, clinical, and imaging findings (for more comprehensive review on the cerebellum and ASD, see Becker and Stoodley, 2013) suggest the cerebellum is part of the distributed neural circuits that are dysfunctional in ASD. Decreased bilateral cerebellar cortical volume has been reported as one of the most important biomarkers for classification of ASD brains (Ecker et al., 2010), and reduction in the posterior midline vermis has been widely shown (e.g., Courchesne et al., 1988, 2011; Kaufmann et al., 2003; Allen et al., 2005). Reduced integrity of the superior cerebellar peduncle (through which output fibers exit the cerebellum) in ASD has also been reported (Sivaswamy et al., 2010) and was associated with degree of social impairment (Catani et al., 2008). Both increases and decreases in cerebellar gray matter (GM) and white matter (WM) have been described in voxel-based morphometry (VBM) studies. Decreased GM is consistently found in midline lobule IX, right Crus I, and lobule VIII in ASD; increased GM has been reported in lobule VI (Cauda et al., 2011; Yu et al., 2011; Duerden et al., 2012; Nickl-Jockschat et al., 2012). These structural differences correlate with scores on autism diagnostic measures: increased GM in lobule VI correlated with worse social and communication scores (Rojas et al., 2006), posterior vermal GM and bilateral Crus II GM correlated with communication scores (Riva et al., 2013), and reduced GM in Crus I was associated with increased repetitive and stereotyped behaviors (Rojas et al., 2006).

ADHD is characterized by behavioral patterns of inattention, hyperactivity, and impulsivity (American Psychiatric Association, 2013). Like ASD and developmental dyslexia, there are several neural systems implicated in ADHD, including frontal-striatal and frontal-cerebellar circuits (for a recent review, see Kasparek et al., 2013). Cerebellar differences are evident on structural, functional, and spectroscopy imaging (Valera et al., 2007), and were the most significant finding in a recent VBM study in medication-naïve adults (Makris et al., 2013). From a pharmacological perspective, the cerebellum is one of the regions that shows altered activation following a single dose of methylphenidate (Czerniak et al., 2013), suggesting that the cerebellar differences are functionally significant in terms of the behavioral profile of ADHD. In the first quantitative study of brain morphometry in ADHD, Castellanos et al. (1996) reported smaller cerebellar volume in ADHD children and adolescents relative to their typically-developing (TD) counterparts. In one of the earliest longitudinal studies investigating neurobiological underpinnings of ADHD, overall cerebellar volume was significantly reduced in ADHD children, a difference that persisted throughout development and correlated with symptom severity (Castellanos et al., 2002). Reduced right cerebellar volume was reported in ADHD children but not their unaffected siblings, even though prefrontal regions showed decreases in both children with ADHD and their unaffected siblings (Durston et al., 2004). As in ASD, posterior vermal volumes (lobules VIII–X) were reduced in ADHD relative to TD comparison groups (with no significant reduction in lobules VI–VII; Berquin et al., 1998; Mostofsky et al., 1998; Castellanos et al., 2001), and the reduction in the posterior vermis correlated with severity of ADHD symptoms (Bledsoe et al., 2011; Ivanov et al., 2014). ADHD children treated with methylphenidate did not show the same decreased volume in the posterior vermis (lobules VIII–X) as untreated ADHD children (Bledsoe et al., 2009), consistent with the proposal that methylphenidate can normalize cerebellar differences in the ADHD brain (see review by Schweren et al., 2013). Though less often reported, Mackie et al. (2007) found that reduced superior cerebellar vermis volume was a stable difference in ADHD children that persisted over time; in the same study, smaller inferior posterior cerebellar hemisphere regions were associated with poorer outcome. VBM studies provide more detailed analyses of cerebellar structure than volumetric measures. Seidman et al. (2011) conducted a region-of-interest VBM study and found reduced GM in several regions in the cerebellum compared to the TD group, including left cerebellar lobules IV–VI, VIII, IX, and X and right cerebellar lobules IV, Crus I, VIII, and IX. Other studies have reported differences in right Crus I (Carmona et al., 2005; Montes et al., 2011), left Crus I (Carmona et al., 2005), and in the posterior vermis (McAlonan et al., 2007; Yang et al., 2008).

Diffusion tensor imaging studies investigating WM differences in ADHD have reported reduced fractional anisotropy in the middle cerebellar peduncles (on the right, Bechtel et al., 2009; on the left, Ashtari et al., 2005), and WM in the left cerebellum (Ashtari et al., 2005; van Ewijk et al., 2013). Functional connectivity studies also suggest differences in cerebellar connectivity in ADHD (Tomasi and Volkow, 2012), particularly in the inattentive subtype (Fair et al., 2013). Reduced cerebellar activation has been reported during working memory tasks in right Crus I (Kobel et al., 2009; Wolf et al., 2009) and left lobule VI (Valera et al., 2005), and reduced functional connectivity was reported in left Crus I and right IX (Wolf et al., 2009).

Developmental dyslexia is defined as deficient literacy skills in the context of normal intelligence and educational opportunity (American Psychiatric Association, 2000). In addition to reading difficulties, poorer performance on a variety of “cerebellar” measures have been reported in dyslexia, including poorer balance, motor skills, and abnormal eye movements (reviewed by Stoodley and Stein, 2011, 2013). The inability of dyslexic readers to achieve fast, fluent reading prompted Nicolson and Fawcett (Nicolson et al., 2001; Nicolson and Fawcett, 2011) to propose the cerebellar theory of dyslexia. They hypothesized that cerebellar dysfunction is a core aspect of the etiology of dyslexia and leads to a deficit in procedural learning, which could explain not only the reading difficulties but also other symptoms associated with dyslexia. Certainly, functional imaging studies show cerebellar engagement during reading tasks (e.g., Turkeltaub et al., 2002; reviewed in Stoodley and Stein, 2011) and a magnetoencephalography study by Kujala et al. (2007) indicated that the cerebellum was one of the forward-driving nodes in the reading network. Structural differences in the cerebellum have been reported in dyslexia (e.g., Eckert, 2004), including differences in symmetry (with dyslexic individuals showing less-asymmetric cerebella than their TD counterparts, who generally show a rightward cerebellar asymmetry; Rae et al., 2002; Kibby et al., 2008; Leonard et al., 2008). Pernet et al. (2009) reported that a region in right lobule VI was the most significant biomarker for classification of adult dyslexic brains.

How might these neural differences arise? Many of the genes implicated in developmental disorders affect brain development at its earliest stages. Of note, many of the candidate genes for these disorders are strongly expressed in the cerebellum; for example, KIAA0319 for dyslexia and CNTNAP2 in ASD (for reviews, see Abrahams and Geschwind, 2010; Carrion-Castillo et al., 2013). Multiple genes have been implicated in each disorder, and linking genetics with imaging data is a relatively new approach (see Durston, 2010). Genetics in ADHD have focused on the dopamine and serotonin systems (e.g., DAT, DRD4, 5-HTT; Faraone et al., 2005), whereas studies of dyslexia have identified several candidate genes that are thought to be involved in early brain development, particularly neuronal migration (e.g., DYX1C1, DCDC2, KIAA0319; for review, Carrion-Castillo et al., 2013). In ASD, a very heterogeneous genetic picture emerges, including multiple candidate genes and a potential significant role of rare copy number variants, with the cerebellum amongst the regions showing a relationship between genetic variants and structural differences in the brain (see Abrahams and Geschwind, 2010, for review). For example, in non-ASD individuals, those who were homozygous for a risk allele on the autism candidate gene CNTNAP2 showed significantly reduced GM in the cerebellum in left lobule VI, bilateral Crus I, and in vermis lobule IX (Tan et al., 2010). It has been hypothesized that shared endophenotypes in these disorders, particularly between ASD and ADHD (see Rommelse et al., 2011), may be due to shared genetic factors (e.g., Rommelse et al., 2010); CNTNAP2, which is expressed in the cerebellum and potentially impacts language function, has been linked to both ASD and dyslexia (Abrahams and Geschwind, 2010; Carrion-Castillo et al., 2013). It is possible that any shared differences in cerebellar structure might be related to some shared genetic factors amongst ASD, ADHD, and dyslexia.

Taken together, these data suggest that in developmental disorders merely stating that there are “cerebellar” findings is perhaps too broad. It is important to consider the location *within* the cerebellum, and the potential for different cerebro-cerebellar circuits to be impacted in different disorders. In other words, are cerebellar findings a general sign of developmental disorder, or are they more specifically related to the etiology of each disorder? Although different regions seem to be involved in ASD, ADHD, and developmental dyslexia, no study has examined this directly. Here we conduct an anatomical likelihood estimate (ALE) meta-analysis of VBM studies of ASD, ADHD, and dyslexia (compared to TD groups). First, we conducted a whole-brain ALE meta-analysis to evaluate the significance of cerebellar differences in the context of the whole brain. Second, we included studies using cerebellar regions of interest for a cerebellar-only analysis in each disorder. Third, we evaluated the overlap of the ALE maps for ASD, ADHD, and dyslexia. Finally, we interpreted our findings in the context of the functional connectivity of the cerebellum with the cerebral cortex, using Buckner et al. (2011)'s 7-network map implemented as part of the Spatially Unbiased Infratentorial (SUIT) cerebellar atlas (Diedrichsen, 2006; Diedrichsen et al., 2009). The findings are discussed in the context of the diagnostic symptoms of each disorder.

## Materials and methods

### Literature search

Articles were identified through a PubMed (http://www.ncbi.nlm.nih.gov/pubmed) search including “autism AND imaging,” “autism AND MRI”; “attention deficit AND imaging,” “attention deficit AND MRI,” “ADHD AND imaging,” “ADHD AND MRI”; and “dyslexia AND imaging,” “dyslexia AND MRI” with the limit “English,” completed in July 2013. Only studies utilizing whole-brain VBM that compared the clinical groups with TD age-matched comparison groups were included. Therefore, for the initial analysis we eliminated studies that reported the results of functional neuroimaging studies; those that reported non-VBM or region-of-interest analyses of structural MRI data; those that did not report the coordinates of the results in standard space (Montreal Neurological Institute, MNI; Collins et al., 1998 or Talairach and Tournoux, 1988); studies reporting incomplete coverage of the cerebellum; and studies that investigated clinical populations without reporting data from a TD comparison group. We also excluded studies whose primary focus was to investigate a comorbid disorder (e.g., individuals with ADHD who were also cocaine-dependent).

The 37 studies listed in Table 1 met these criteria. There were 17 VBM studies studying ASD, 10 investigating ADHD, and 10 for developmental dyslexia. These analyses thus represent the data from a total of 363 ASD participants vs. 373 TD controls; 249 ADHD participants vs. 248 TD controls; and 173 dyslexic participants vs. 163 TD controls. Twenty-one studies investigated children and adolescents (8 ASD, 8 ADHD, and 5 dyslexia studies) and adults were the participants in 14 studies (7 ASD, 2 ADHD, and 5 dyslexia studies).

**Table 1:** **Studies included in the analyses**.

	**Study by author**	**Total *N***	**Clinical group *N* (gender)**	**TD group *N* (gender)**	**Age (years) clinical group**	**Age (years) TD group**
ASD	Abell et al., 1999	30	15 (12 males)	15 (12 males)	28.8	25.3
	Boddaert et al., 2004	33	21 (16 males)	12 (7 males)	9.3	10.8
	Bonilha et al., 2008	28	12 (12 males)	16 (16 males)	12.4	13.2
	Brieber et al., 2007	30	15 (15 males)	15 (15 males)	Adults	Adults
	Craig et al., 2007	33	14 (0 males)	19 (0 males)	37.9	35
	Ecker et al., 2010^*^	44	22 (22 males)	22 (22 males)	27	28
	Ecker et al., 2012	178	89 (89 males)	89 (89 males)	26	28
	Hyde et al., 2010	30	15 (15 males)	15 (15 males)	22.7	19.2
	Ke et al., 2008	32	17 (14 males)	15 (12 males)	8.9	9.7
	Kwon et al., 2004	33	20 (20 males)	13 (13 males)	13.5	13.6
	McAlonan et al., 2005	34	17 (16 males)	17 (16 males)	11	12
	McAlonan et al., 2008	88	33 (27 males)	55 (47 males)	11.6	10.7
	Riva et al., 2011	42	21 (13 males)	21 (13 males)	6.5	6.8
	Rojas et al., 2006	47	24 (24 males)	23 (23 males)	20.8	21.4
	Schmitz et al., 2006	22	10 (10 males)	12 (12 males)	36.0	38.0
	Waiter et al., 2004	32	16 (16 males)	16 (16 males)	15.4	15.5
	Wilson et al., 2009	20	10 (8 males)	10 (7 males)	30.1	29.4
	Total ASD	736	363 (323 males)	373 (326 males)	19.0 years (10.1 *SD*)	18.7 years (9.2 *SD*)
ADHD	Ahrendts et al., 2010	40	31 (20 males)	31 (20 males)	31.2	31.5
	Brieber et al., 2007	30	15 (15 males)	15 (15 males)	13.1	13.3
	Carmona et al., 2005	50	25 (21 males)	25 (21 males)	10.8	11.2
	Kobel et al., 2010	26	14 (14 males)	12 (12 males)	10.4	10.9
	Lim et al., 2013	58	29 (29 males)	29 (29 males)	13.8	14.4
	McAlonan et al., 2007	59	28 (28 males)	31 (31 males)	9.9	9.6
	Overmeyer et al., 2001	34	18 (15 males)	16 (15 males)	10.4	10.3
	Sasayama et al., 2010	35	18 (13 males)	17 (12 males)	10.6	10.0
	van Wingen et al., 2013	29	14 (14 males)	15 (15 males)	32.0	37.0
	Yang et al., 2008	114	57 (35 males)	57 (34 males)	11.1	11.7
	Total ADHD	497	249 (204 males)	248 (204 males)	15.3 years (8.7 *SD*)	16.0 years (9.8 *SD*)
Dyslexia	Brambati et al., 2004	21	10 (5 males)	11 (5 males)	31.6	27.4
	Brown et al., 2001	30	16 (16 males)	14 (14 males)	24	24
	Eckert et al., 2005	26	13 (13 males)	13 (13 males)	11.4	11.3
	Hoeft et al., 2007	38	19 (10 males)	19 (10 males)	14.4	14.4
	Jednorog et al., 2013	81	46 (26 males)	35 (13 males)	10.3	10.3
	Kronbichler et al., 2008	28	13 (13 males)	15 (15 males)	15.9	15.5
	Silani et al., 2005	64	32 (32 males)	32 (32 males)	24.4	26.3
	Siok et al., 2008	32	16 (8 males)	16 (13 males)	11.0	11.0
	Steinbrink et al., 2008	16	8 (6 males)	8 (6 males)	20.1	23.7
	Vinckenbosch et al., 2005	23	13 (13 males)	10 (10 males)	Adults	Adults
	Total Dyslexia	336	173 (142 males)	163 (121 males)	18.1 (7.4 *SD*)	18.2 (7.1 *SD*)

* *This study used support vector machine (SVM) analysis of VBM data*.

### Cerebellar region of interest analysis

Since our goal was to focus on cerebellar structural differences in ASD, ADHD, and dyslexia, we also conducted a secondary analysis in which only cerebellar coordinates were included in the ALE analysis. Studies not reporting cerebellar differences were not included in this analysis, and therefore not all studies included in the whole-brain analysis were added to the cerebellum-only analysis. In this analysis, we also included studies which conducted voxel-based analyses in which cerebellar findings were reported as part of a region of interest (ROI) analysis. Five additional studies were included (2 for ASD, 2 for ADHD, 1 for dyslexia). All studies included in the ROI analysis are listed in Table 2. As with most ROI analyses, limiting the coordinates included in the analysis to the cerebellum increases statistical power for detecting voxel-level differences within the ROI.

**Table 2:** **Studies included in the cerebellar ROI analyses**.

	**Study by author**	**Total *N***	**Clinical group *N* (gender)**	**TD group *N* (gender)**	**Age (years) clinical group**	**Age (years) TD group**
ASD	Ecker et al., 2010	44	22 (22 males)	22 (22 males)	27	28
	Ecker et al., 2012	178	89 (89 males)	89 (89 males)	26	28
	McAlonan et al., 2005	34	17 (16 males)	17 (16 males)	11	12
	McAlonan et al., 2008	88	33 (27 males)	55 (47 males)	11.6	10.7
	Riva et al., 2011	42	21 (13 males)	21 (13 males)	6.5	6.8
	Rojas et al., 2006	47	24 (24 males)	23 (23 males)	20.8	21.4
	Salmond et al., 2005	27	14 (13 males)	13 (13 males)	12.9	12.1
	Salmond et al., 2007	44	22 (20 males)	22 (19 males)	11.8	12.1
	Wilson et al., 2009	20	10 (8 males)	10 (7 males)	30.1	29.4
ADHD	Carmona et al., 2005	50	25 (21 males)	25 (21 males)	10.8	11.2
	Lim et al., 2013	58	29 (29 males)	29 (29 males)	13.8	14.4
	McAlonan et al., 2007	59	28 (28 males)	31 (31 males)	9.9	9.6
	Montes et al., 2011^*^	23	11 (0 males)	12 (0 males)	7.2	7.8
	Montes et al., 2011^*^	18	8 (0 males)	10 (0 males)	14.9	14.9
	Montes et al., 2011^*^	20	10 (0 males)	10 (0 males)	27.9	26.5
	Seidman et al., 2011	128	74 (38 males)	54 (25 males)	37.3	34.3
	Yang et al., 2008	114	57 (35 males)	57 (34 males)	11.1	11.7
Dyslexia	Brambati et al., 2004	21	10 (5 males)	11 (5 males)	31.6	27.4
	Brown et al., 2001	30	16 (16 males)	14 (14 males)	24	24
	Eckert et al., 2005	26	13 (13 males)	13 (13 males)	11.4	11.3
	Kronbichler et al., 2008	28	13 (13 males)	15 (15 males)	15.9	15.5
	Pernet et al., 2009	77	38 (34 males)	39 (35 males)	27.2	27.8

* *This study separately compared children, adolescents, and adults with ADHD vs. age-matched TD populations, and thus was entered as three separate comparisons in the ALE analysis*.

### Co-morbid disorders in included studies

While we excluded studies which focused on co-morbid disorders, it is possible that within each sample there were participants with, for example, ADHD and developmental dyslexia. It is less likely that there would be a co-morbid group of ASD and ADHD participants, as the versions of the Diagnostic and Statistical Manual of Mental Disorders (DSM) that were employed in these studies excluded symptoms of inattention and hyperactivity “during the course of a pervasive developmental disorder,” which by nature excludes ASD from the ADHD diagnosis (e.g., American Psychiatric Association, 2000).

In ADHD, the most commonly reported co-morbid disorders were anxiety disorder (Carmona et al., 2005; McAlonan et al., 2007; Yang et al., 2008; Kobel et al., 2010), major depressive disorder (Seidman et al., 2011), conduct disorder (Overmeyer et al., 2001; McAlonan et al., 2007; Kobel et al., 2010; Sasayama et al., 2010), oppositional defiant disorder (Overmeyer et al., 2001; Yang et al., 2008; Sasayama et al., 2010), and obsessive-compulsive disorder (McAlonan et al., 2007; Kobel et al., 2010). These disorders are commonly co-morbid with ADHD (Biederman, 2005). Only one study reported a participant with co-morbid dyslexia (Overmeyer et al., 2001; one participant in their clinical sample of 18). Two studies noted a subset of participants with learning disability. In Seidman et al. (2011) the diagnosis of co-morbid learning disability in their adult sample (9 adults of 74 ADHD participants) was based on scores on reading and/or arithmetic scores (Wide Range Achievement Test), and so could include participants with reading disorder; in Yang et al. (2008), 5 of the 57 ADHD children were diagnosed with learning disability. In each study, the number of participants with learning disability (which may or may not include reading disability) was relatively low.

In the ASD group, the vast majority of studies ruled out genetic syndromes such as Fragile X as part of their standard exclusion criteria. In the one study in which there was a Fragile X comparison group (Wilson et al., 2009), we only included the data from the ASD group without Fragile X. In the one study including both ASD and ADHD participants (Brieber et al., 2007), the individuals with ASD did not have symptoms of ADHD and *vice versa*. Three other ASD studies specifically noted that ADHD was an exclusion criterion (Salmond et al., 2005, 2007; McAlonan et al., 2008), though most studies more broadly excluded psychiatric disorders. In the dyslexia group, the diagnosis of reading disorder excludes reading difficulties that could be better attributed to another disorder. Several studies did specifically note that individuals with co-morbid ADHD were excluded (Brown et al., 2001; Vinckenbosch et al., 2005; Jednorog et al., 2013).

### Data extraction and analysis

#### Anatomic likelihood estimate (ALE) meta-analysis

The ALE meta-analysis method for imaging studies was originally described by Turkeltaub et al. (2002). This method treats each focus as the center of a probability distribution, rather than a single point, and so is better able to deal with inevitable inter-study differences in scanning parameters and imaging analyses (Turkeltaub et al., 2002). Newer iterations of the program (GingerALE software 2.3, www.brainmap.org/ale; Eickhoff et al., 2009, 2012; Turkeltaub et al., 2012) incorporate random effects analysis and a modification to limit the effect of any single experiment on the ALE results (Turkeltaub et al., 2012).

The meta-analysis procedure using GingerALE is summarized as follows. Text files for each clinical group (ASD, ADHD, dyslexia) were generated that contained the GM foci reported in each study for the clinical group vs. TD group comparison, with separate files for clinical group > TD and clinical group < TD. Foci in Talairach space were converted to MNI space using the icbm2tal transform (Lancaster et al., 2007) prior to analysis. Foci that were reported in Talairach space that had been transformed from MNI space using the Brett transform were converted back to MNI space using the Brett transform (mni2tal) rather than icbm2tal. The foci were blurred with a full-width half-maximum (FWHM) calculated based on the subject size of each study. GingerALE uses the foci from each study to create a Modeled Activation (MA) map for each study by taking the maximum across each focus' Gaussian (Turkeltaub et al., 2012). The union of all the MA maps creates the ALE image. The null distribution of the ALE statistic at each voxel was determined as described in Eickhoff et al. (2012). ALE maps were thresholded at *p* < 0.001 (uncorrected) with a minimum cluster size (*k*) of 50 for the whole-brain analyses and at false discovery rate (FDR)-corrected *p* < 0.01 (*k* > 50) for the cerebellum-only ROI analysis. The data were visualized using MRIcron (http://www.mccauslandcenter.sc.edu/mricro/mricron/) with the thresholded ALE maps as the overlay, and the SUIT (Diedrichsen, 2006; Diedrichsen et al., 2009) template as the underlay.

#### Anatomical localization of results

The GingerALE program outputs the size, extent, weighted center, peak coordinates, and ALE values for each cluster. We used the SUIT atlas (Diedrichsen, 2006; Diedrichsen et al., 2009) to localize the cluster peak coordinates to different lobules of the cerebellum.

#### Network analyses

In order to evaluate the cerebellar findings in the context of cerebro-cerebellar circuits, we utilized the Buckner et al. (2011) 7-network map available in MRIcron in conjunction with the SUIT cerebellar atlas. The 7-network map is the result of a winner-takes-all algorithm at each voxel in the cerebellum used to determine which of 7 cortical networks (defined by Yeo et al., 2011) showed the strongest functional correlation with that cerebellar region. These data resulted from functional connectivity analyses in 1000 healthy adults (500 in the discovery sample, 500 in the replication sample). Figure 1C shows the connectivity map and the corresponding 7 cortical networks, including the somatomotor, visual, limbic, frontoparietal, dorsal and ventral attention, and default mode networks. We overlaid our thresholded ALE maps on top of the cerebellar connectivity maps in order to estimate the functional connectivity of the cerebellar regions showing significant effects in each disorder.

## Results

### ASD

**Figure 2:**
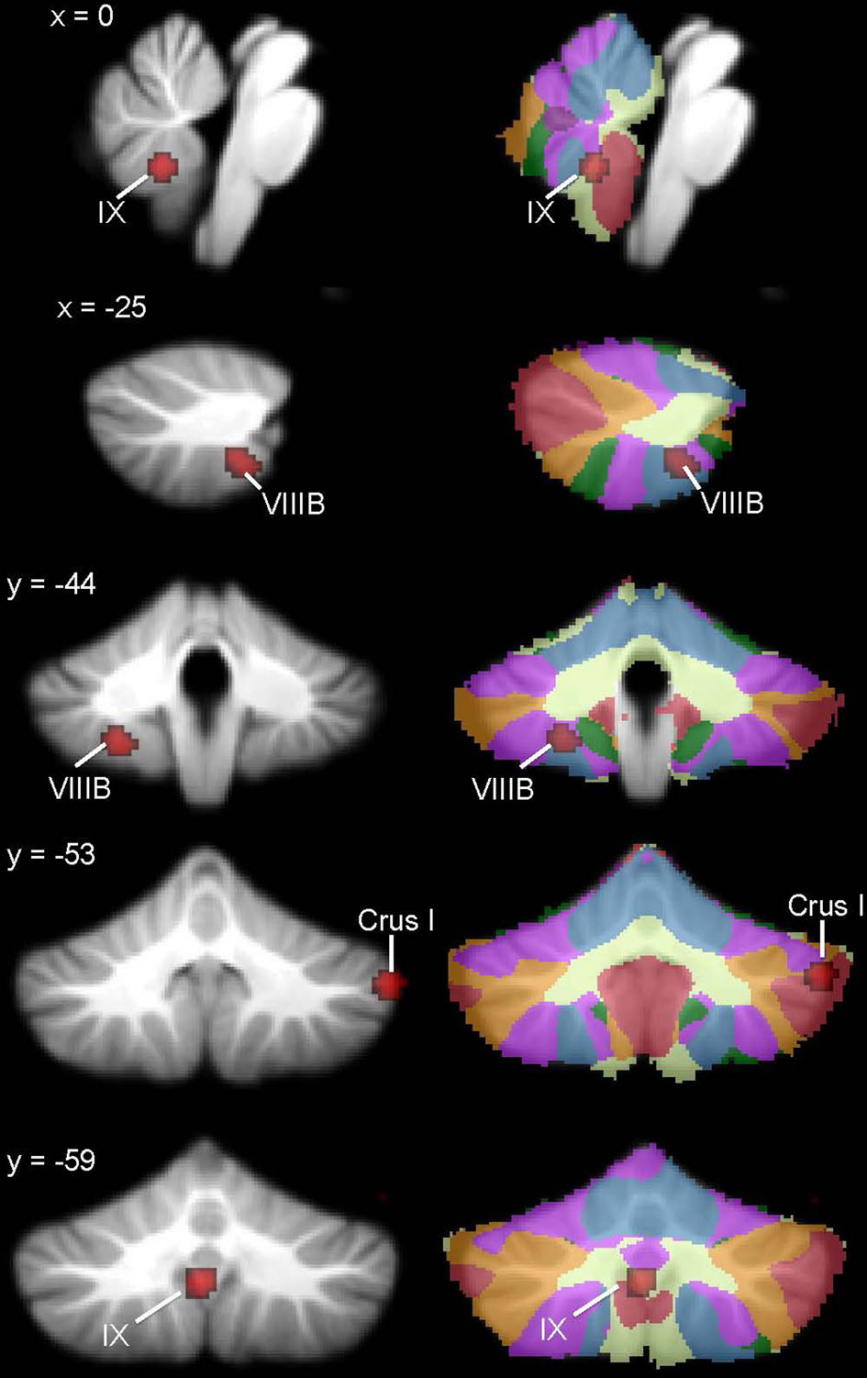
**Cerebellar GM differences in ASD. Left**, regions in the whole-brain analysis showing significant ALE voxels where ASD < TD (red), thresholded at *p* < 0.001, *k* > 50. **Right**, corresponding slices showing functional connectivity maps of the cerebellum (Buckner et al., 2011). Networks are color-coded such that blue, somatomotor; green, dorsal attention; violet, ventral attention; cream, limbic; orange, frontoparietal; red, default network.

**Figure 3:**
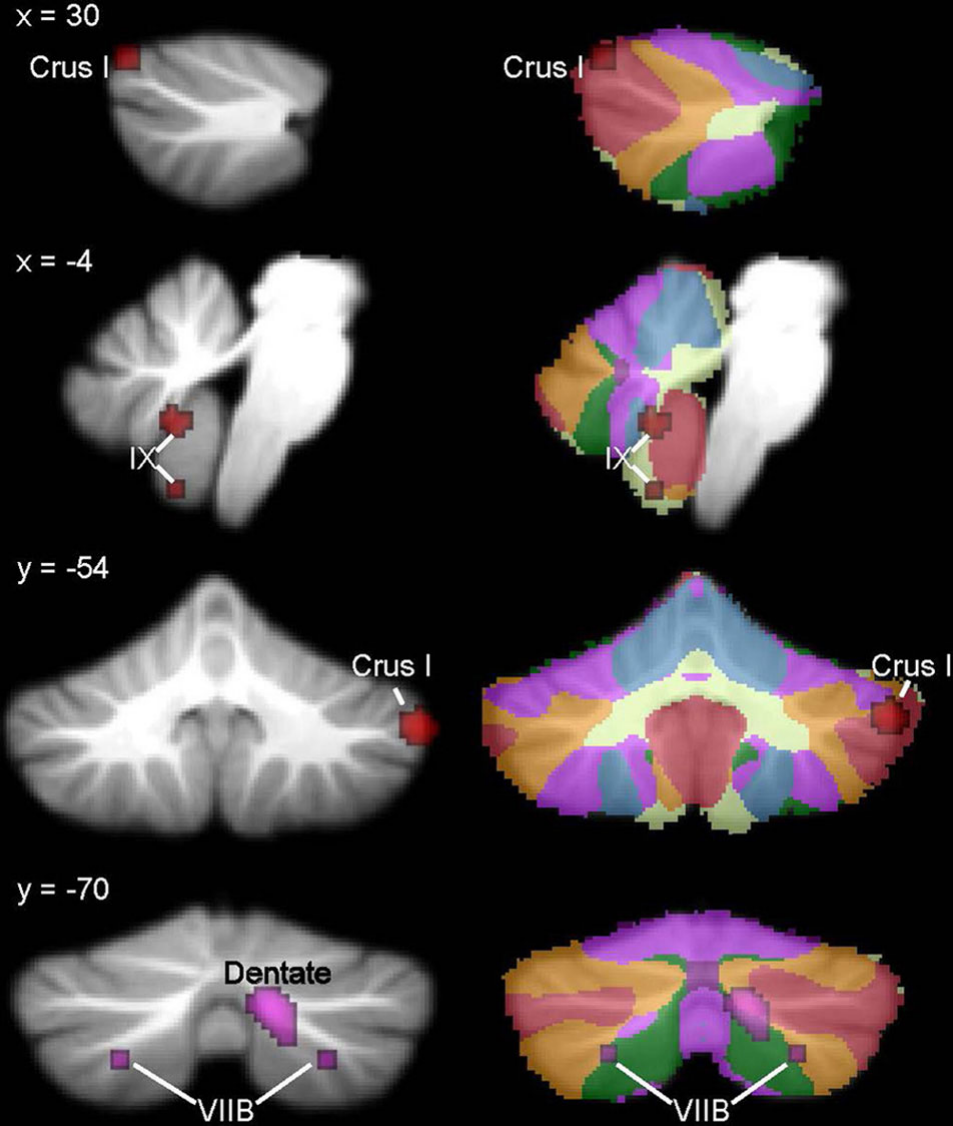
**ASD cerebellar ROI analysis**. Left, ASD < TD in additional IX and right Crus I clusters (red) and regions where ASD > TD (violet) in the right dentate nucleus and bilaterally in VIIB. These ALE maps are thresholded at FDR-corrected *p* < 0.05, *k* > 50. Right, corresponding slices showing functional connectivity maps of the cerebellum (Buckner et al., 2011). Networks are color-coded such that blue, somatomotor; green, dorsal attention; violet, ventral attention; cream, limbic; orange, frontoparietal; red, default network.

The whole brain analysis revealed significant GM reduction in cerebellar clusters in right Crus I, left lobule VIIIB, and vermis lobule IX in ASD (Figure 2, Table 3; whole-brain results are available in Supplementary Table 1: the most significant cluster in the brain was in the left cuneus [BA 7], followed by the left cerebellar VIII cluster, the right Crus I cluster, and the midline IX cluster). No GM increases were found in the cerebellum. The cerebellar-only ALE analysis (Figure 3, Table 3), including the additional studies with cerebellar ROIs, revealed GM reduction again in right Crus I, midline IX, and left VIIIB, with more voxels reaching significance than in the whole-brain analysis. The cerebellar analysis of GM increases showed increased GM in the right dentate nucleus and bilaterally in lobule VIIB.

**Table 3:** **GM differences in ASD: whole brain and cerebellar-only analyses**.

	**Cluster number**	**Volume (mm^3^)**	**ALE value × 10^−3^**	***x***	***y***	***z***	**Location**
**WHOLE BRAIN ANALYSIS**							
ASD < TD	1	368	11.50	−24	−44	−52	Left VIIIB
	2	352	13.98	54	−54	−36	Right Crus I
	3	328	12.85	−2	−60	−40	Midline IX
	4	56	10.41	30	−84	−24	Right Crus I
ASD>TD	No sig. clusters						
**CEREBELLAR ROI ANALYSIS**							
ASD < TD	1	416	13.98	54	−54	−36	Right Crus I
	2	400	13.13	0	−58	−40	Midline IX
	3	152	10.41	30	−84	−24	Right Crus I
	4	64	7.87	−4	−60	−58	Midline IX
	5	56	9.02	−26	−48	−48	Left VIIIB
ASD > TD	1	784	12.96	12	−68	−36	Right dentate
	2	72	8.41	26	−72	−50	Right VIIB
	3	64	8.35	−26	−72	−48	Left VIIB

*ASD, Autism spectrum disorder; TD, typically-developing; ROI, region of interest; x, y, z, MNI coordinates*.

### ADHD

**Figure 4:**
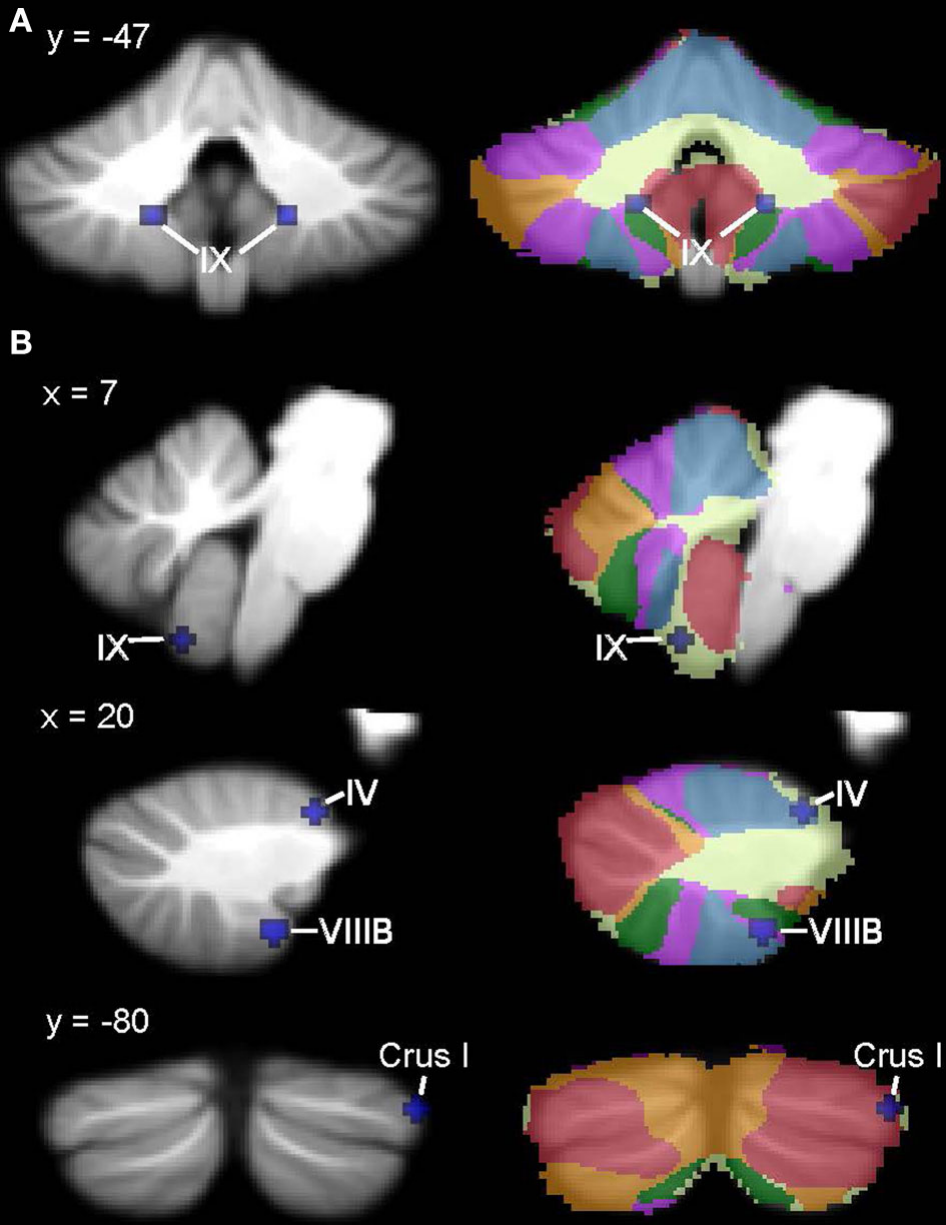
**ADHD < TD GM: whole-brain and ROI analyses**. Left **(A)**, Regions in the whole-brain analysis showing significant ALE voxels where ADHD < TD (blue), thresholded at *p* < 0.001, *k* > 50. **(B)** Regions showing ADHD < TD in the ROI analysis. Right, corresponding slices showing functional connectivity maps of the cerebellum (Buckner et al., 2011). Networks are color-coded such that blue, somatomotor; green, dorsal attention; violet, ventral attention; cream, limbic; orange, frontoparietal; red, default network.

In the ADHD group, significant ALE values indicating decreased GM were found in bilateral cerebellar lobule IX (Figure 4, Table 4; whole-brain results are reported in Supplementary Table 2). Clusters in the basal ganglia (putamen, caudate), medial frontal gyrus (BA 11), cuneus (BA 18), and amygdala had more significant ALE values than the cerebellar clusters (see Supplementary Table 2). There were no cerebellar regions in which increased GM was found relative to the TD groups. In the cerebellar ROI analysis, in addition to the bilateral IX clusters reported as part of the whole-brain analysis, significant GM differences (ADHD < TD) were found in right Crus I, left lobule X, and bilaterally in VIIIB.

**Table 4:** **GM differences in ADHD: whole brain and cerebellar-only analyses**.

	**Cluster number**	**Volume (mm^3^)**	**ALE value × 10^−3^**	***x***	***y***	***z***	**Location**
**WHOLE-BRAIN ANALYSIS**							
ADHD < TD	1	80	9.67	−16	−48	−45	Left lobule IX
	2	64	8.99	18	−48	−46	Right lobule IX
**CEREBELLAR ROI ANALYSIS**							
ADHD<TD	1	96	10.49	20	−44	−54	Right VIIIB
	2	80	9.80	−16	−48	−46	Left lobule IX
	3	72	9.75	18	−46	−46	Right lobule IX
	4	64	10.21	56	−68	−32	Right Crus I
	5	56	9.98	6	−60	−58	Right lobule IX
	6	56	10.03	−14	−44	−56	Left VIIIB
	7	56	9.99	−26	−32	−40	Left X
	8	56	9.98	44	−80	−30	Right Crus I
	9	56	9.98	20	−34	−24	Right I–IV

*ADHD, attention deficit hyperactivity disorder; TD, typically-developing; ROI, region of interest; x, y, z, MNI coordinates*.

### Developmental dyslexia

**Figure 5:**
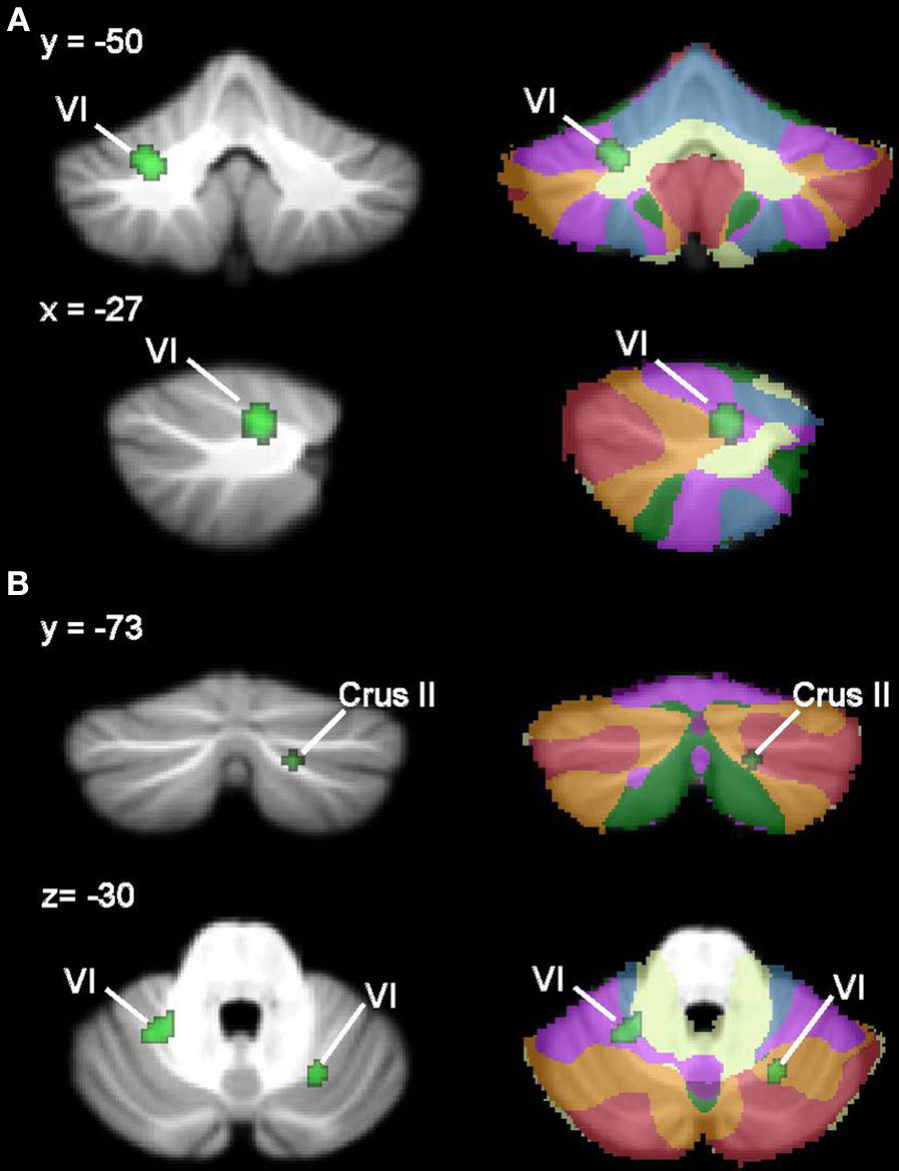
**Dyslexia < TD GM: whole-brain and ROI analyses**. Left **(A)**, Regions in the whole-brain analysis showing significant ALE voxels where Dyslexia < TD (green), thresholded at *p* < 0.001, *k* > 50. **(B)** Regions showing Dyslexia < TD in the ROI analysis. Right, corresponding slices showing functional connectivity maps of the cerebellum (Buckner et al., 2011). Networks are color-coded such that blue, somatomotor; green, dorsal attention; violet, ventral attention; cream, limbic; orange, frontoparietal; red, default network.

ALE analysis of VBM studies in developmental dyslexia only revealed regions in which GM in dyslexia was reduced relative to TD groups (Figure 5, Table 5; whole-brain analyses are in Supplementary Table 3). In dyslexia, the most significant and largest ALE cluster in the whole brain was in the cerebellum (left lobule VI); a second significant cluster was found in right lobule VI. In addition to the bilateral lobule VI clusters, the cerebellar ROI analyses revealed regions of reduced GM in dyslexia in right lobule VI and right Crus II.

**Table 5:** **GM differences in dyslexia: whole brain and cerebellar-only analyses**.

	**Cluster number**	**Volume (mm^3^)**	**ALE value × 10^−3^**	***x***	***y***	***z***	**Location**
**WHOLE-BRAIN ANALYSIS**							
Dyslexia < TD	1	392	10.18	−26	−50	−32	Left VI
	2	64	6.73	32	−52	−20	Right VI
**CEREBELLAR ROI ANALYSIS**							
Dyslexia < TD	1	304	8.98	−26	−50	−30	Left VI
	2	208	9.72	26	−64	−28	Right VI
	3	64	7.13	26	−54	−32	Right VI
	4	56	7.14	18	−74	−38	Right Crus II

*TD, typically-developing; ROI, region of interest; x, y, z, MNI coordinates*.

### Convergence or divergence?

**Figure 6:**
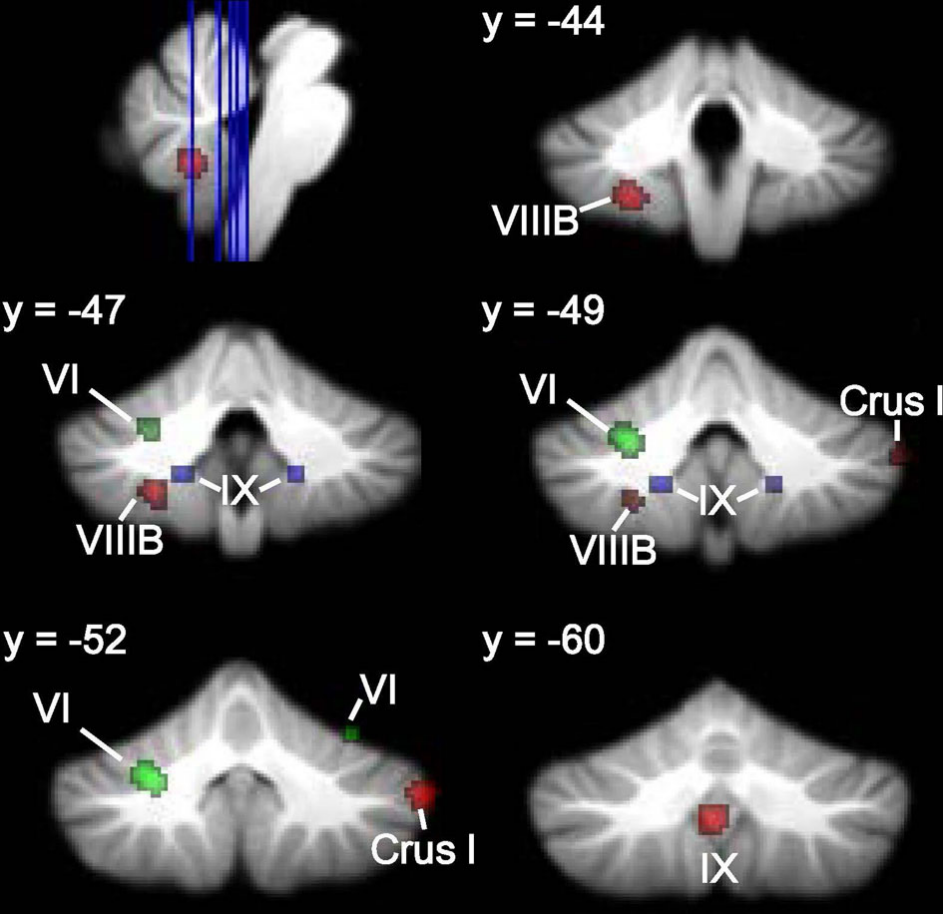
**Different cerebellar regions affected in ASD vs. ADHD vs. dyslexia**. Coronal slices through the cerebellum show clusters of GM decreases in ASD (red), ADHD (blue), and dyslexia (green).

**Figure 7:**
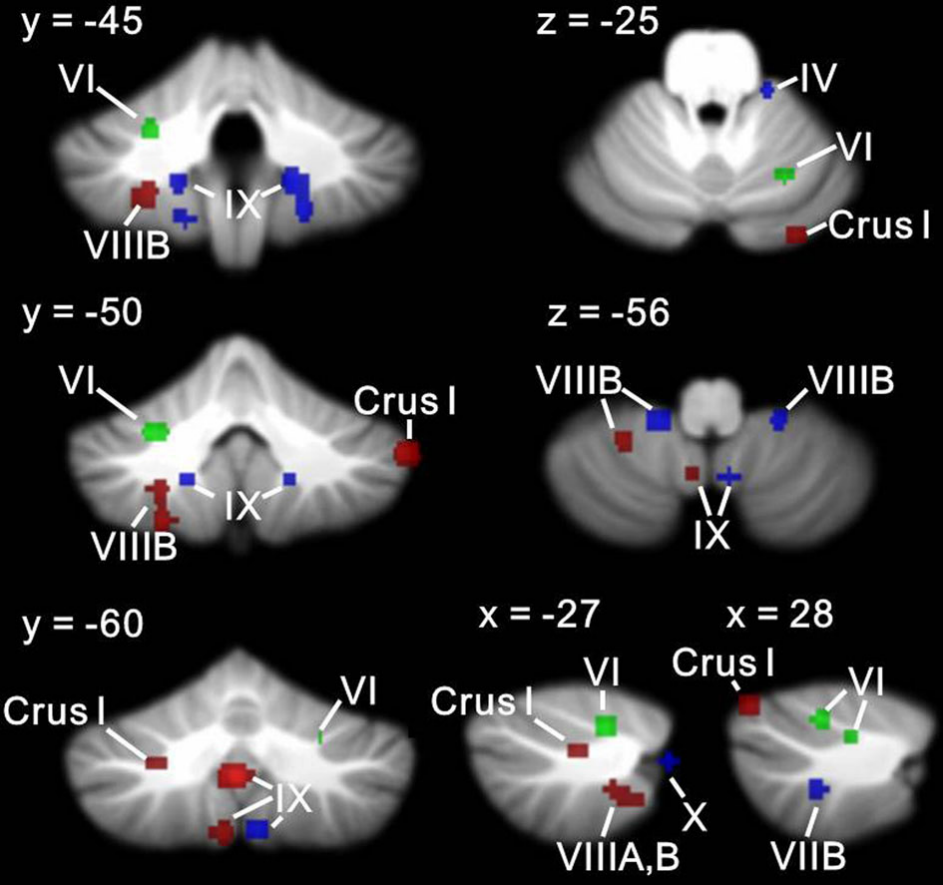
**Different cerebellar regions affected in ASD vs. ADHD vs. dyslexia in the cerebellar ROI analyses**. Coronal, axial, and sagittal slices through the cerebellum show clusters of GM decreases in ASD (red), ADHD (blue), and dyslexia (green) resulting from the ROI analysis.

Figures 6, 7 show the ALE maps of the locations where the clinical groups have less GM than the TD groups for ASD, ADHD, and dyslexia. It is clear, based on the whole-brain analyses (Figure 6), that different regions of the cerebellum are impacted in each disorder. Even though the ROI analyses revealed additional significant cerebellar clusters within each group, there were no overlapping cerebellar regions affected (Figure 7).

### Network analysis

We estimated the functional significance of the cerebellar findings in ASD, ADHD, and dyslexia using Buckner et al.'s (2011) 7-network functional connectivity mapping of the cerebellum, available in conjunction with the SUIT cerebellar atlas. This functional connectivity map enables us to evaluate which of 7 broad cortical networks shows the strongest functional connectivity with each of our cerebellar clusters. The 7 cortical networks include the somatomotor, frontoparietal, dorsal and ventral attention, visual, limbic, and default mode networks (see Buckner et al., 2011 and Yeo et al., 2011, for more details).

In ASD, the right Crus I cluster fell within both the frontoparietal cortical network and the default mode network. The left lobule VIIIB cluster mapped to the somatomotor network, whereas the vermis lobule IX cluster mapped to the limbic network. Figure 2 (right) shows these clusters in the context of the functional connectivity mapping of the cerebellum. Additional clusters from the ROI analysis were also in the limbic (additional IX cluster) and default (second right Crus I cluster) networks (Figure 3, right). In ADHD, the clusters were part of the dorsal attention network (Figure 4, right); the additional clusters emerging from the cerebellar ROI analysis involved the ventral attention network (additional bilateral VIIIB clusters), the somatomotor network (right anterior lobe), and the default network (right Crus I cluster). In dyslexia, the left VI cluster was located in the part of the cerebellum mapping to the ventral attention network (Figure 5, right); the clusters identified in the cerebellar ROI analysis mapped to the frontoparietal (right VI) and default mode networks (right Crus II). Therefore, while there were no regions of overlap within the cerebellum anatomically, there is some degree of overlap as to the potential cerebro-cerebellar networks affected in these disorders.

## Discussion

Our ALE meta-analysis of VBM studies revealed that different regions of the cerebellum show reduced GM in ASD, ADHD, and developmental dyslexia relative to TD age-matched individuals. The cerebellar findings were significant in the context of whole-brain analyses, and were supported and extended by the cerebellar ROI analyses. The network analyses also suggested that different cerebro-cerebellar networks are disrupted in these developmental disorders, and it is possible that, within each disorder, different core symptoms are mediated by unique cerebro-cerebellar circuits. This is consistent with the repeating, modular circuitry of the cerebellar cortex, such that discrete sub-regions within the cerebellum have different functions. In this context, cerebellar modulation could be applied to various cortical networks, including somatomotor, attention, frontoparietal, and default mode networks (see Ito, 2008). These findings indicate that the localization of regional differences in ASD, ADHD, and dyslexia and their functional significance should be considered in the context of the functional topography of the human cerebellum.

In ASD, three regions of reduced GM emerged from the analysis: midline lobule IX, right Crus I, and left lobule VIIIB; in functional connectivity analyses, these regions are associated with limbic, frontoparietal/default mode, and somatomotor networks, respectively (Buckner et al., 2011). Therefore, the structural and functional connectivity of these regions are consistent with the complex behavioral profile of ASD, which includes difficulties in social interaction and communication, as well as repetitive and stereotyped behaviors. Structurally, the midline lobule IX cluster is consistent with early anatomical reports of reduced volume of the inferior cerebellar vermis in ASD (e.g., Courchesne et al., 1988, 1994). In terms of the functional significance of this region, vermal damage in preterm infants has also been associated with ASD symptoms (Limperopoulos et al., 2008). Clinical studies investigating the behavioral effects of cerebellar malformations also support a relationship between vermal malformations and positive ASD screens, whereas cerebellar hemisphere malformations are more often associated with selective deficits in executive function, language, or spatial cognition (Tavano et al., 2007; Bolduc et al., 2011, 2012). Right Crus I is active during cognitive tasks, including language and working memory paradigms (e.g., Stoodley and Schmahmann, 2009), and right posterolateral damage to the cerebellum has been associated with language deficits (e.g., Riva and Giorgi, 2000; Stoodley et al., 2012a). It is therefore possible that right Crus I differences could be related to communication deficits in ASD. The relationship between this right Crus I cluster and the frontoparietal association network supports this idea, as does a recent report showing reduced functional connectivity between right Crus I and cortical language regions including the supplementary motor area, inferior frontal gyrus, and dorsolateral prefrontal cortex in ASD (Verly et al., 2014). The right Crus I cluster also overlapped with cerebellar regions associated with the default mode network, which is thought to be dysfunctional in ASD (see review by Minshew and Keller, 2010) and is associated with various aspects of social processing (Li et al., 2014). Consistent with our structural finding, a large resting-state functional connectivity study reported reduced functional connectivity in the cerebellum and the default mode networks in ASD (Tomasi and Volkow, 2012). Crus I is also engaged during empathy and theory of mind tasks in TD individuals (Vollm et al., 2006), which suggests that structural differences in this region may contribute to the well-documented difficulties with empathy and theory of mind in ASD individuals. Finally, we found reduced GM in left lobule VIIIB in ASD, which may be related to impaired motor control (see Becker and Stoodley, 2013, for review of motor symptoms in ASD), since lobule VIIIB participates in somatomotor networks (Krienen and Buckner, 2009; Buckner et al., 2011) and is active during sensorimotor tasks (e.g., Stoodley and Schmahmann, 2009, for review). The finding that cerebellar differences in ASD map to different functional regions of the cerebellum—including those that are involved in affective function, language, and motor control—suggests that GM reductions in midline lobule IX, right Crus I, and left VIIIB may make different contributions to the core symptoms of ASD.

In ADHD, the whole-brain analysis showed GM reduction bilaterally in lobule IX; notably, not the same regions of IX as were found in ASD. Instead, these clusters mapped to cerebellar regions associated with the dorsal attention network (Buckner et al., 2011), which is thought to modulate voluntary allocation of attention (see Corbetta and Shulman, 2002; Vossel et al., 2014, for review). In the ROI analysis, GM reductions were also found in VIIIB and right Crus I, in regions involving the ventral attention network and default mode network, respectively. A cluster was also found in the anterior lobe of the cerebellum, which shows structural and functional connectivity with sensorimotor areas of the cerebral cortex (see Krienen and Buckner, 2009; Stoodley and Schmahmann, 2010). Frontal-cerebellar circuits have been associated with timing functions that are hypothesized to be crucial to prediction and attention (Ghajar and Ivry, 2009), and timing is considered a critical impairment in ADHD (e.g., Sonuga-Barke et al., 2010). Consistent with this, Durston et al. (2011) have proposed that frontal-cerebellar circuits in ADHD are specifically related to timing, a concept which is supported by a range of neuroimaging studies reporting cerebellar dysfunction in ADHD during time duration judgments and violations of timing (e.g., Durston et al., 2007; Valera et al., 2010; Vloet et al., 2010). The functional connectivity of the cerebellar regions associated with ADHD with the dorsal and ventral attention networks and the default mode network make sense in the context of the networks thought to be affected in ADHD (Bush, 2010, for review). Consistent with the decreased GM in VIIIB found in the ROI analysis, Hoekzema et al. (2010, 2011) found that VIIIB showed GM and BOLD signal increases following cognitive training in children with ADHD, which was associated with improved performance on an attention task. These findings suggest that the cerebellum mediates attention-related circuitry that is relevant to the etiology of ADHD.

In participants with developmental dyslexia, reduced GM was located in lobule VI bilaterally; when the ROI analysis was performed, an additional cluster was found in right Crus II. These findings are consistent with other studies in dyslexia, including Pernet et al.'s (2009) report that a right lobule VI cluster was the best biomarker for differentiating adults with dyslexia. The left lobule VI cluster was associated with the ventral attention network, whereas the right VI cluster mapped to the frontoparietal network, and the right Crus II cluster to the default mode network. While the default mode network has not been explicitly investigated in dyslexia, dyslexia is thought to involve dysfunction in a left-lateralized reading network (for recent review, see Richlan, 2012), which involves regions associated with both the frontoparietal and ventral attention networks. Although the predominant causal theory of dyslexia proposes a core deficit in phonological processing (e.g., Bradley and Bryant, 1983)—which may be relevant to the right lobule VI cluster, which connects with left cerebral cortex—it has also been proposed that dyslexia results from deficient visual-spatial attention (Vidyasagar and Pammer, 2010), which could be related to the left lobule VI cluster, projecting to right-lateralized cortical regions. The idea that visual-spatial attention is important to reading development is supported by a recent study investigating predictors of reading achievement in preschoolers, which found that a visual attention task was a better predictor of later reading skills than naming and language tasks (Franceschini et al., 2012). The ventral attention network includes the right temporo-parietal junction (TPJ; Corbetta and Shulman, 2002), and neuroimaging evidence suggests significant overactivation of this area during both phonological (Simos et al., 2000; Richlan et al., 2009) and attention tasks (Goldfarb and Shaul, 2013) in dyslexic participants. Our finding that the most significant cerebellar ALE cluster in dyslexia was associated with the ventral attention network is consistent with these data. The right lobule VI cluster is consistent with right-lateralized activation in the cerebellum for language tasks (Stoodley and Schmahmann, 2009), and reduced GM here may be relevant to phonological processing difficulties in dyslexia. As to the specific contribution of the cerebellum to reading (see Stoodley and Stein, 2011, 2013, for reviews), the observation that dyslexic readers very rarely achieve fluent, automatic reading led to the proposal that an impairment in cerebellar procedural learning functions could lead to the range of symptoms seen in dyslexia (Nicolson et al., 2001; Nicolson and Fawcett, 2007, 2011). Consistent with the idea that modification of cerebellar circuits could improve skill acquisition, cerebellar GM in the right anterior lobe increased in response to successful remediation in dyslexia (Krafnick et al., 2011). The left VI cerebellar cluster identified here overlaps with regions of overactivation in dyslexia during functional imaging studies (Linkersdorfer et al., 2012). The authors suggest that the overactivation in this region reflects the increased effort or compensatory strategies of dyslexic readers during reading tasks. In summary, as in ASD, it is possible that the different cerebellar clusters showing GM alterations in dyslexia may make differential contributions to the behavioral manifestations of the disorder, such that the left lobule VI cluster is involved in visual-spatial attention difficulties and the right lobule VI cluster is related to phonological deficits. Consistent with this idea, left lobule VI was involved in spatial processing while right lobule VI was involved in language and working memory processing in a meta-analysis of task-based neuroimaging studies (Stoodley and Schmahmann, 2009).

### Divergent cerebellar structural findings across disorders and co-morbidity

The results of this ALE meta-analysis suggest that different regions of the cerebellum show GM reductions in ASD, ADHD, and developmental dyslexia. Although there were no regions of overlap between the disorders, there were some commonalities in the putative cerebro-cerebellar networks affected, including the ventral attention network for ADHD and dyslexia, and the frontoparietal and default networks for ASD and dyslexia. This is not surprising, given the co-morbidities between these disorders: it is estimated that ~40% of individuals with ADHD have reading difficulties (e.g., Willcutt et al., 2010), ~30% of males with ADHD have elevated ASD traits (Reiersen et al., 2007), and ~30% of individuals with ASD show clinically significant signs of ADHD (e.g., Simonoff et al., 2008). Imaging studies have been attempting to establish the unique and shared neural substrates between ASD and ADHD, using both functional (task-based, Christakou et al., 2013; functional connectivity, Di Martino et al., 2013) and structural (Brieber et al., 2007) approaches. The only relevant cerebellar findings thus far revealed overactivation in ASD relative to ADHD and TD groups in the anterior left cerebellum during a sustained attention task (Christakou et al., 2013). In ADHD and dyslexia, structural imaging has focused on specific regions of interest (Hynd et al., 1990; Semrud-Clikeman et al., 1996; Kibby et al., 2009a) and overall cerebral volume (Kibby et al., 2009b), with no cerebellar findings to report. Future prospective structural and functional imaging studies will be required to examine the relationship between cerebellar structure and function in ASD, ADHD, and dyslexia and the overlapping symptoms among these disorders.

### Limitations

In any meta-analysis of neuroimaging data, there are inherent limitations due to combining studies using different groups of participants (with different ages, diagnostic criteria, co-morbidities, medication status), imaging parameters and analytical approaches, including normalization methods and thresholding. The ALE approach attempts to accommodate issues related to imaging analyses and registration by treating each focus as the center of a Gaussian probability distribution, and foci are converted between Talairach and MNI space using algorithms that depend on the initial processing software (e.g., SPM vs. FSL). However, there are limitations to the ALE approach, such that it does not account for differences in cluster size between studies. We tried to limit the effect of these issues by restricting our analyses to whole-brain VBM studies which directly compared the patient groups with age-matched TD groups. The strength of this approach is that we were able to determine regions of the cerebellum which are consistently reported in such studies, pooling data from a larger number of participants. Another limitation is the use of the 7-network cerebellar map (Buckner et al., 2011) to interpret the predominant cerebral networks associated with our cerebellar clusters. As these maps only include 7 networks, and are based on a winner-takes-all algorithm, there are surely nuances in functional connectivity that will be missed with this approach. Therefore, the results of this study should be used as a basis for future prospective investigations of the role(s) of different cerebellar regions in ASD, ADHD, and developmental dyslexia.

Another limitation is that the results of VBM studies cannot determine the cellular alterations that produce the observed differences in GM (see Zatorre et al., 2012). That said, molecular analyses of animal models and post-mortem studies in ASD, ADHD, and dyslexia can provide some clues. The cerebellar cortex is comprised of three layers (the molecular layer, Purkinje cell layer, and the granule cell layer); inputs come in via the mossy fibers and climbing fibers, and the sole output neurons of the cerebellar cortex are the Purkinje cells, which are amongst the largest neurons in the brain with extensively branched dendritic trees. The most substantial body of post-mortem data comes from ASD, where smaller Purkinje cell size and reduced number and density of Purkinje cells have been reported (reviewed in Becker and Stoodley, 2013). These findings suggest that GM differences in ASD might be related to these alterations in Purkinje cells, although Kemper and Bauman (1993) also reported reduced numbers of granule cells in the cerebellum in ASD brains. There are very few, if any, post-mortem analyses evaluating the cerebellum for ADHD and developmental dyslexia. One study investigating a small sample of 4 dyslexic brains reported differences in Purkinje cell area (significantly larger in the posterior lobe) and cell density (a trend toward reduced cell density in the posterior lobe), consistent with our findings of reduced GM in the posterior lobe of the cerebellum in dyslexia. In a rodent model of ADHD, a reduced number of Purkinje cells in the cerebellar vermis was reported, which was not present in the ADHD rats treated with methylphenidate (Yun et al., 2014). Finally, a recent study investigating GAD65 antibodies in the serum of children with ASD and ADHD found that the serum of 60% of ASD and 53% of ADHD participants reacted with cerebellar Purkinje cells; in a smaller subset of ADHD participants (20%) the serum also reacted with cells in the molecular and granule cell layers (Rout et al., 2012). The authors suggest a potential relationship between the Purkinje cell loss reported in ASD and GAD65 reactivity in their ASD group (Rout et al., 2012); this relationship may also be present in ADHD. While only a small body of evidence, these data suggest that the differences in GM reported here may have a basis in cellular differences in the circuitry of the cerebellar cortex.

### Future directions

The results of this meta-analysis suggest that different cerebellar regions are affected in ASD, ADHD, and dyslexia. Further, the clusters where anatomical differences were found in the whole-brain analysis are associated with different functional circuits which are consistent with the behavioral profiles of each disorder: e.g., the default mode network in ASD, the dorsal attention network in ADHD. These findings indicate that the specific sub-region of the cerebellum that is affected in a given developmental disorder should be considered in the context of cerebellar functional topography and cerebro-cerebellar connectivity. Future studies will aim to determine how and when these cerebellar differences arise in the context of neural development, and the specific contribution that cerebellar dysfunction makes to the behavioral manifestations of autism, ADHD, and developmental dyslexia.

### Conflict of interest statement

The author declares that the research was conducted in the absence of any commercial or financial relationships that could be construed as a potential conflict of interest.

## References

[B1] Abell F. Krams M. Ashburner J. Passingham R. Friston K. Frackowiak R. (1999). The neuroanatomy of autism: a voxel-based whole brain analysis of structural scans. Neuroreport 10, 1647–1651 10.1097/00001756-199906030-00005 10501551

[B2] Abrahams B. S. Geschwind D. H. (2010). Connecting genes to brain in the autism spectrum disorders. Arch. Neurol. 67, 395–399 10.1001/archneurol.2010.47 20385903 PMC3645845

[B3] Ahrendts J. Rusch N. Wilke M. Philipsen A. Eickhoff S. B. Glauche V. (2010). Visual cortex abnormalities in adults with ADHD: a structural MRI study. World J. Biol. Psychiatry 12, 260–270 10.3109/15622975.2010.518624 20879808

[B4] Allen G. McColl R. Barnard H. Ringe W. Fleckenstein J. Cullum C. (2005). Magnetic resonance imaging of cerebellar-prefrontal and cerebellar parietal functional connectivity. Neuroimage 28, 39–48 10.1016/j.neuroimage.2005.06.013 16023375

[B5] American Psychiatric Association (2000). Diagnostic and Statistical Manual of Mental Disorders, 4th Edn., Text revision. Washington, DC: American Psychiatric Association 10.1176/appi.books.9780890423349

[B6] American Psychiatric Association (2013). Diagnostic and Statistical Manual of Mental Disorders, 5th Edn. Arlington, VA: American Psychiatric Association, Inc

[B7] Apps R. Garwicz M. (2005). Anatomical and physiological foundations of cerebellar information processing. Nat. Rev. Neurosci. 6, 297–311 10.1038/nrn1646 15803161

[B8] Ashtari M. Kumra S. Bhaskar S. L. Clarke T. Thaden E. Cervellione K. L. (2005). Attention-deficit/hyperactivity disorder: a preliminary diffusion tensor imaging study. Biol. Psychiatry 57, 448–455 10.1016/j.biopsych.2004.11.047 15737658

[B9] Bailey A. Luthert P. Dean A. Harding B. Janota I. Montgomery M. (1998). A clinicopathological study of autism. Brain 121(Pt 5), 889–905 10.1093/brain/121.5.889 9619192

[B10] Bauman M. Kemper T. (1985). Histoanatomic observations of the brain in early infantile autism. Neurology 35, 866–874 10.1212/WNL.35.6.866 4000488

[B11] Bechtel N. Kobel M. Penner I. K. Klarhofer M. Scheffler K. Opwis K. (2009). Decreased fractional anisotropy in the middle cerebellar peduncle in children with epilepsy and/or attention deficit/hyperactivity disorder: a preliminary study. Epilepsy Behav. 15, 294–298 10.1016/j.yebeh.2009.04.005 19362604

[B12] Becker E. B. Stoodley C. J. (2013). Autism spectrum disorder and the cerebellum. Int. Rev. Neurobiol. 113, 1–34 10.1016/B978-0-12-418700-9.00001-0 24290381

[B13] Berquin P. C. Giedd J. N. Jacobsen L. K. Hamburger S. D. Krain A. L. Rapoport J. L. (1998). Cerebellum in attention-deficit hyperactivity disorder-A morphometric MRI study. Neurology 50, 1087–1093 10.1212/WNL.50.4.1087 9566399

[B14] Biederman J. (2005). Attention-deficit/hyperactivity disorder: a selective overview. Biol. Psychiatry 57, 1215–1220 10.1016/j.biopsych.2004.10.020 15949990

[B15] Bledsoe J. Semrud-Clikeman M. Pliszka S. R. (2009). A magnetic resonance imaging study of the cerebellar vermis in chronically treated and treatment-naive children with attention-deficit/hyperactivity disorder combined type. Biol. Psychiatry 65, 620–624 10.1016/j.biopsych.2008.11.030 19150052 PMC2675536

[B16] Bledsoe J. C. Semrud-Clikeman M. Pliszka S. R. (2011). Neuroanatomical and neuropsychological correlates of the cerebellum in children with attention-deficit/hyperactivity disorder–combined type. J. Am. Acad. Child Adolesc. Psychiatry 50, 593–601 10.1016/j.jaac.2011.02.014 21621143 PMC3104210

[B17] Boddaert N. Chabane N. Gervais H. Good C. D. Bourgeois M. Plumet M. H. (2004). Superior temporal sulcus anatomical abnormalities in childhood autism: a voxel-based morphometry MRI study. Neuroimage 23, 364–369 10.1016/j.neuroimage.2004.06.016 15325384

[B18] Bolduc M. E. Du Plessis A. J. Evans A. Guizard N. Zhang X. Robertson R. L. (2011). Cerebellar malformations alter regional cerebral development. Dev. Med. Child Neurol. 53, 1128–1134 10.1111/j.1469-8749.2011.04090.x 22066826 PMC3220736

[B19] Bolduc M. E. du Plessis A. J. Sullivan N. Guizard N. Zhang X. Robertson R. L. (2012). Regional cerebellar volumes predict functional outcome in children with cerebellar malformations. Cerebellum 11, 531–542 10.1007/s12311-011-0312-z 21901523

[B20] Bonilha L. Cendes F. Rorden C. Eckert M. Dalgalarrondo P. Li L. M. (2008). Gray and white matter imbalance–typical structural abnormality underlying classic autism? Brain Dev. 30, 396–401 10.1016/j.braindev.2007.11.006 18362056

[B21] Bradley L. Bryant P. (1983). Categorising sounds and learning to read–a causal connection. Nature 301, 419–421 10.1038/301419a0

[B22] Brambati S. M. Termine C. Ruffino M. Stella G. Fazio F. Cappa S. F. (2004). Regional reductions of gray matter volume in familial dyslexia. Neurology 63, 742–745 10.1212/01.WNL.0000134673.95020.EE 15326259

[B23] Brieber S. Neufang S. Bruning N. Kamp-Becker I. Remschmidt H. Herpertz-Dahlmann B. (2007). Structural brain abnormalities in adolescents with autism spectrum disorder and patients with attention deficit/hyperactivity disorder. J. Child Psychol. Psychiatry 48, 1251–1258 10.1111/j.1469-7610.2007.01799.x 18093031

[B24] Brown W. Eliez S. Menon V. Rumsey J. White C. Reiss A. (2001). Preliminary evidence of widespread morphological variations in the brain in dyslexia. Neurology 56, 781–783 10.1212/WNL.56.6.781 11274316

[B25] Buckner R. L. Krienen F. M. Castellanos A. Diaz J. C. Yeo B. T. (2011). The organization of the human cerebellum estimated by intrinsic functional connectivity. J. Neurophysiol. 106, 2322–2345 10.1152/jn.00339.2011 21795627 PMC3214121

[B26] Bush G. (2010). Attention-deficit/hyperactivity disorder and attention networks. Neuropsychopharmacology 35, 278–300 10.1038/npp.2009.120 19759528 PMC3055423

[B27] Carmona S. Vilarroya O. Bielsa A. Tremols V. Soliva J. C. Rovira M. (2005). Global and regional gray matter reductions in ADHD: a voxel-based morphometric study. Neurosci. Lett. 389, 88–93 10.1016/j.neulet.2005.07.020 16129560

[B27a] Carrion-Castillo A. Franke B. Fisher S. E. (2013). Molecular genetics of dyslexia: an overview. Dyslexia 19, 214–240 10.1002/dys.1464 24133036

[B28] Castellanos F. Lee P. Sharp W. Jeffries N. Greenstein D. Clasen L. (2002). Developmental trajectories of brain volume abnormalities in children and adolescents with attention-deficit/hyperactivity disorder. JAMA 288, 1740–1748 10.1001/jama.288.14.1740 12365958

[B29] Castellanos F. X. Giedd J. N. Berquin P. C. Walter J. M. Sharp W. Tran T. (2001). Quantitative brain magnetic resonance imaging in girls with attention-deficit/hyperactivity disorder. Arch. Gen. Psychiatry 58, 289–295 10.1001/archpsyc.58.3.289 11231836

[B30] Castellanos F. X. Giedd J. N. Marsh W. L. Hamburger S. D. Vaituzis A. C. Dickstein D. P. (1996). Quantitative brain magnetic resonance imaging in attention-deficit hyperactivity disorder. Arch. Gen. Psychiatry 53, 607–616 10.1001/archpsyc.1996.01830070053009 8660127

[B31] Catani M. Jones D. Daly E. Embiricos N. Deeley Q. Pugliese L. (2008). Altered cerebellar feedback projections in Asperger syndrome. Neuroimage 41, 1184–1191 10.1016/j.neuroimage.2008.03.041 18495494

[B32] Cauda F. Geda E. Sacco K. D'Agata F. Duca S. Geminiani G. (2011). Grey matter abnormality in autism spectrum disorder: an activation likelihood estimation meta-analysis study. J. Neurol. Neurosurg. Psychiatry 82, 1304–1313 10.1136/jnnp.2010.239111 21693631

[B33] Christakou A. Murphy C. M. Chantiluke K. Cubillo A. I. Smith A. B. Giampietro V. (2013). Disorder-specific functional abnormalities during sustained attention in youth with Attention Deficit Hyperactivity Disorder (ADHD) and with autism. Mol. Psychiatry 18, 236–244 10.1038/mp.2011.185 22290121 PMC3554878

[B34] Collins D. Zijdenbos A. Kollokian V. Sled J. Kabani N. Holmes C. (1998). Design and construction of a realistic digital brain phantom. IEEE Trans. Med. Imaging 17, 463–468 10.1109/42.712135 9735909

[B35] Corbetta M. Shulman G. L. (2002). Control of goal-directed and stimulus-driven attention in the brain. Nat. Rev. Neurosci. 3, 201–215 10.1038/nrn755 11994752

[B36] Courchesne E. Campbell K. Solso S. (2011). Brain growth across the life span in autism: age-specific changes in anatomical pathology. Brain Res. 1380, 138–145 10.1016/j.brainres.2010.09.101 20920490 PMC4500507

[B37] Courchesne E. Saitoh O. Townsend J. P. Yeung-Courchesne R. Press G. A. Lincoln A. J. (1994). Cerebellar hypoplasia and hyperplasia in infantile autism. Lancet 343, 63–64 10.1016/S0140-6736(94)90923-7 7905084

[B38] Courchesne E. Yeung-Courchesne R. Press G. Hesselink J. Jernigan T. (1988). Hypoplasia of cerebellar vermal lobules VI and VII in autism. New Engl. J. Med. 318, 1349–1354 10.1056/NEJM198805263182102 3367935

[B39] Craig M. C. Zaman S. H. Daly E. M. Cutter W. J. Robertson D. M. Hallahan B. (2007). Women with autistic-spectrum disorder: magnetic resonance imaging study of brain anatomy. Br. J. Psychiatry 191, 224–228 10.1192/bjp.bp.106.034603 17766762

[B40] Czerniak S. M. Sikoglu E. M. King J. A. Kennedy D. N. Mick E. Frazier J. (2013). Areas of the brain modulated by a single-dose methylphenidate treatment in youth with ADHD during task-based fMRI: a systematic review. Harv. Rev. Psychiatry 21, 151–162 10.1097/HRP.0b013e318293749e 23660970 PMC4103657

[B41] Diedrichsen J. (2006). A spatially unbiased atlas template of the human cerebellum. Neuroimage 33, 127–138 10.1016/j.neuroimage.2006.05.056 16904911

[B42] Diedrichsen J. Balsters J. H. Flavell J. Cussans E. Ramnani N. (2009). A probabilistic MR atlas of the human cerebellum. Neuroimage 46, 39–46 10.1016/j.neuroimage.2009.01.045 19457380

[B43] Di Martino A. Zuo X. N. Kelly C. Grzadzinski R. Mennes M. Schvarcz A. (2013). Shared and distinct intrinsic functional network centrality in autism and attention-deficit/hyperactivity disorder. Biol. Psychiatry 74, 623–632 10.1016/j.biopsych.2013.02.011 23541632 PMC4508007

[B44] Duerden E. G. Mak-Fan K. M. Taylor M. J. Roberts S. W. (2012). Regional differences in grey and white matter in children and adults with autism spectrum disorders: an activation likelihood estimate (ALE) meta-analysis. Autism Res. 5, 49–66 10.1002/aur.235 22139976

[B45] Durston S. (2010). Imaging genetics in ADHD. Neuroimage 52, 832–838 10.1016/j.neuroimage.2010.02.071 20206707

[B46] Durston S. Davidson M. C. Mulder M. J. Spicer J. A. Galvan A. Tottenham N. (2007). Neural and behavioral correlates of expectancy violations in attention-deficit hyperactivity disorder. J. Child Psychol. Psychiatry 48, 881–889 10.1111/j.1469-7610.2007.01754.x 17714373

[B47] Durston S. Hulshoff Pol H. E. Schnack H. G. Buitelaar J. K. Steenhuis M. P. Minderaa R. B. (2004). Magnetic resonance imaging of boys with attention-deficit/hyperactivity disorder and their unaffected siblings. J. Am. Acad. Child Adolesc. Psychiatry 43, 332–340 10.1097/00004583-200403000-00016 15076267

[B48] Durston S. van Belle J. de Zeeuw P. (2011). Differentiating frontostriatal and fronto-cerebellar circuits in attention-deficit/hyperactivity disorder. Biol. Psychiatry 69, 1178–1184 10.1016/j.biopsych.2010.07.037 20965496

[B49] Ecker C. Rocha-Rego V. Johnston P. Mourao-Miranda J. Marquand A. Daly E. M. (2010). Investigating the predictive value of whole-brain structural MR scans in autism: a pattern classification approach. Neuroimage 49, 44–56 10.1016/j.neuroimage.2009.08.024 19683584

[B50] Ecker C. Suckling J. Deoni S. C. Lombardo M. V. Bullmore E. T. Baron-Cohen S. (2012). Brain anatomy and its relationship to behavior in adults with autism spectrum disorder: a multicenter magnetic resonance imaging study. Arch. Gen. Psychiatry 69, 195–209 10.1001/archgenpsychiatry.2011.1251 22310506

[B51] Eckert M. (2004). Neuroanatomical markers for dyslexia: a review of dyslexia structural imaging studies. Neuroscientist 10, 362–371 10.1177/1073858404263596 15271263

[B52] Eckert M. A. Leonard C. M. Wilke M. Eckert M. Richards T. Richards A. (2005). Anatomical signatures of dyslexia in children: unique information from manual and voxel based morphometry brain measures. Cortex 41, 304–315 10.1016/S0010-9452(08)70268-5 15871596

[B53] Eickhoff S. B. Bzdok D. Laird A. R. Kurth F. Fox P. T. (2012). Activation likelihood estimation meta-analysis revisited. Neuroimage 59, 2349–2361 10.1016/j.neuroimage.2011.09.017 21963913 PMC3254820

[B54] Eickhoff S. B. Laird A. R. Grefkes C. Wang L. E. Zilles K. Fox P. T. (2009). Coordinate-based activation likelihood estimation meta-analysis of neuroimaging data: a random-effects approach based on empirical estimates of spatial uncertainty. Hum. Brain Mapp. 30, 2907–2926 10.1002/hbm.20718 19172646 PMC2872071

[B55] Fair D. A. Nigg J. T. Iyer S. Bathula D. Mills K. L. Dosenbach N. U. (2013). Distinct neural signatures detected for ADHD subtypes after controlling for micro-movements in resting state functional connectivity MRI data. Front. Syst. Neurosci. 6:80 10.3389/fnsys.2012.00080 23382713 PMC3563110

[B56] Faraone S. V. Perlis R. H. Doyle A. E. Smoller J. W. Goralnick J. J. Holmgren M. A. (2005). Molecular genetics of attention-deficit/hyperactivity disorder. Biol. Psychiatry 57, 1313–1323 10.1016/j.biopsych.2004.11.024 15950004

[B57] Franceschini S. Gori S. Ruffino M. Pedrolli K. Facoetti A. (2012). A causal link between visual spatial attention and reading acquisition. Curr. Biol. 22, 814–819 10.1016/j.cub.2012.03.013 22483940

[B58] Ghajar J. Ivry R. B. (2009). The predictive brain state: asynchrony in disorders of attention? Neuroscientist 15, 232–242 10.1177/1073858408326429 19074688 PMC4342364

[B59] Goldfarb L. Shaul S. (2013). Abnormal attentional internetwork link in dyslexic readers. Neuropsychology 27, 725–729 10.1037/a0034422 24040923

[B60] Habas C. Kamdar N. Nguyen D. Prater K. Beckmann C. F. Menon V. (2009). Distinct cerebellar contributions to intrinsic connectivity networks. J. Neurosci. 29, 8586–8594 10.1523/JNEUROSCI.1868-09.2009 19571149 PMC2742620

[B61] Hoeft F. Meyler A. Hernandez A. Juel C. Taylor-Hill H. Martindale J. L. (2007). Functional and morphometric brain dissociation between dyslexia and reading ability. Proc. Natl. Acad. Sci. U.S.A. 104, 4234–4239 10.1073/pnas.0609399104 17360506 PMC1820738

[B62] Hoekzema E. Carmona S. Ramos-Quiroga J. A. Barba E. Bielsa A. Tremols V. (2011). Training-induced neuroanatomical plasticity in ADHD: a tensor-based morphometric study. Hum. Brain Mapp. 32, 1741–1749 10.1002/hbm.21143 21365715 PMC6870061

[B63] Hoekzema E. Carmona S. Tremols V. Gispert J. D. Guitart M. Fauquet J. (2010). Enhanced neural activity in frontal and cerebellar circuits after cognitive training in children with attention-deficit/hyperactivity disorder. Hum. Brain Mapp. 31, 1942–1950 10.1002/hbm.20988 20336653 PMC6871170

[B64] Hyde K. L. Samson F. Evans A. C. Mottron L. (2010). Neuroanatomical differences in brain areas implicated in perceptual and other core features of autism revealed by cortical thickness analysis and voxel-based morphometry. Hum. Brain Mapp. 31, 556–566 10.1002/hbm.20887 19790171 PMC6870833

[B65] Hynd G. W. Semrud-Clikeman M. Lorys A. R. Novey E. S. Eliopulos D. (1990). Brain morphology in developmental dyslexia and attention deficit disorder/hyperactivity. Arch. Neurol. 47, 919–926 10.1001/archneur.1990.00530080107018 2375699

[B66] Ito M. (2008). Control of mental activities by internal models in the cerebellum. Nat. Rev. Neurosci. 9, 304–313 10.1038/nrn2332 18319727

[B67] Ivanov I. Murrough J. W. Bansal R. Hao X. Peterson B. S. (2014). Cerebellar morphology and the effects of stimulant medications in youths with attention deficit-hyperactivity disorder. Neuropsychopharmacology 39, 718–726 10.1038/npp.2013.257 24077064 PMC3895250

[B68] Jednorog K. Gawron N. Marchewka A. Heim S. Grabowska A. (2013). Cognitive subtypes of dyslexia are characterized by distinct patterns of grey matter volume. Brain Struct. Funct. [Epub ahead of print]. 10.1007/s00429-013-0595-6 23775490 PMC4147248

[B69] Kasparek T. Theiner P. Filova A. (2013). Neurobiology of ADHD from childhood to adulthood: findings of imaging methods. J. Atten. Disord. [Epub ahead of print]. 10.1177/1087054713505322 24097847

[B70] Kaufmann W. E. Cooper K. L. Mostofsky S. H. Capone G. T. Kates W. R. Newschaffer C. J. (2003). Specificity of cerebellar vermian abnormalities in autism: a quantitative magnetic resonance imaging study. J. Child Neurol. 18, 463–470 10.1177/08830738030180070501 12940651

[B71] Ke X. Hong S. Tang T. Zou B. Li H. Hang Y. (2008). Voxel-based morphometry study on brain structure in children with high-functioning autism. Neuroreport 19, 921–925 10.1097/WNR.0b013e328300edf3 18520994

[B72] Kemper T. L. Bauman M. L. (1993). The contribution of neuropathologic studies to the understanding of autism. Neurol. Clin. 11, 175–187 8441369

[B73] Kibby M. Y. Fancher J. B. Markanen R. Hynd G. W. (2008). A quantitative magnetic resonance imaging analysis of the cerebellar deficit hypothesis of dyslexia. J. Child Neurol. 23, 368–380 10.1177/0883073807309235 18160557 PMC2440485

[B74] Kibby M. Y. Kroese J. M. Krebbs H. Hill C. E. Hynd G. W. (2009a). The pars triangularis in dyslexia and ADHD: a comprehensive approach. Brain Lang. 111, 46–54 10.1016/j.bandl.2009.03.001 19356794 PMC2759398

[B75] Kibby M. Y. Pavawalla S. P. Fancher J. B. Naillon A. J. Hynd G. W. (2009b). The relationship between cerebral hemisphere volume and receptive language functioning in dyslexia and attention-deficit hyperactivity disorder (ADHD). J. Child Neurol. 24, 438–448 10.1177/0883073808324772 19211921 PMC2664863

[B76] Kobel M. Bechtel N. Specht K. Klarhofer M. Weber P. Scheffler K. (2010). Structural and functional imaging approaches in attention deficit/hyperactivity disorder: does the temporal lobe play a key role? Psychiatry Res. 183, 230–236 10.1016/j.pscychresns.2010.03.010 20702071

[B77] Kobel M. Bechtel N. Weber P. Specht K. Klarhofer M. Scheffler K. (2009). Effects of methylphenidate on working memory functioning in children with attention deficit/hyperactivity disorder. Eur. J. Paediatr. Neurol. 13, 516–523 10.1016/j.ejpn.2008.10.008 19056305

[B78] Krafnick A. J. Flowers D. L. Napoliello E. M. Eden G. F. (2011). Gray matter volume changes following reading intervention in dyslexic children. Neuroimage 57, 733–741 10.1016/j.neuroimage.2010.10.062 21029785 PMC3073149

[B79] Krienen F. M. Buckner R. L. (2009). Segregated fronto-cerebellar circuits revealed by intrinsic functional connectivity. Cereb. Cortex 19, 2485–2497 10.1093/cercor/bhp135 19592571 PMC2742600

[B80] Kronbichler M. Wimmer H. Staffen W. Hutzler F. Mair A. Ladurner G. (2008). Developmental dyslexia: gray matter abnormalities in the occipitotemporal cortex. Hum. Brain Mapp. 29, 613–625 10.1002/hbm.20425 17636558 PMC6871168

[B81] Kujala J. Pammer K. Cornelissen P. Roebroeck A. Formisano E. Salmelin R. (2007). Phase coupling in a cerebro-cerebellar network at 8-13 Hz during reading. Cereb. Cortex 17, 1476–1485 10.1093/cercor/bhl059 16926241

[B82] Kwon H. Ow A. W. Pedatella K. E. Lotspeich L. J. Reiss A. L. (2004). Voxel-based morphometry elucidates structural neuroanatomy of high-functioning autism and Asperger syndrome. Dev. Med. Child Neurol. 46, 760–764 10.1111/j.1469-8749.2004.tb00996.x 15540637

[B83] Lancaster J. L. Tordesillas-Gutierrez D. Martinez M. Salinas F. Evans A. Zilles K. (2007). Bias between MNI and Talairach coordinates analyzed using the ICBM-152 brain template. Hum. Brain Mapp. 28, 1194–1205 10.1002/hbm.20345 17266101 PMC6871323

[B84] Leonard C. M. Kuldau J. M. Maron L. Ricciuti N. Mahoney B. Bengtson M. (2008). Identical neural risk factors predict cognitive deficit in dyslexia and schizophrenia. Neuropsychology 22, 147–158 10.1037/0894-4105.22.2.147 18331156

[B85] Levinson H. N. (1990). The diagnostic value of cerebellar-vestibular tests in detecting learning disabilities, dyslexia, and attention deficit disorder. Percept. Mot. Skills 71, 67–82 10.2466/pms.1990.71.1.67 2235277

[B86] Li W. Mai X. Liu C. (2014). The default mode network and social understanding of others: what do brain connectivity studies tell us. Front. Hum. Neurosci. 8:74 10.3389/fnhum.2014.00074 24605094 PMC3932552

[B87] Lim L. Marquand A. Cubillo A. A. Smith A. B. Chantiluke K. Simmons A. (2013). Disorder-specific predictive classification of adolescents with attention deficit hyperactivity disorder (ADHD) relative to autism using structural magnetic resonance imaging. PLoS ONE 8:e63660 10.1371/journal.pone.0063660 23696841 PMC3656087

[B88] Limperopoulos C. Bassan H. Sullivan N. R. Soul J. S. Robertson R. L. Jr. Moore M. (2008). Positive screening for autism in ex-preterm infants: prevalence and risk factors. Pediatrics 121, 758–765 10.1542/peds.2007-2158 18381541 PMC2703587

[B89] Linkersdorfer J. Lonnemann J. Lindberg S. Hasselhorn M. Fiebach C. J. (2012). Grey matter alterations co-localize with functional abnormalities in developmental dyslexia: an ALE meta-analysis. PLoS ONE 7:e43122 10.1371/journal.pone.0043122 22916214 PMC3423424

[B90] Mackie S. Shaw P. Lenroot R. Pierson R. Greenstein D. K. Nugent T. F. (2007). Cerebellar development and clinical outcome in attention deficit hyperactivity disorder. Am. J. Psychiatry 164, 647–655 10.1176/appi.ajp.164.4.647 17403979

[B91] Makris N. Liang L. Biederman J. Valera E. M. Brown A. B. Petty C. (2013). Toward defining the neural substrates of ADHD: a controlled structural MRI study in medication-naive adults. J. Atten. Disord. [Epub ahead of print]. 10.1177/1087054713506041 24189200 PMC4009385

[B92] McAlonan G. M. Cheung V. Cheung C. Chua S. E. Murphy D. G. Suckling J. (2007). Mapping brain structure in attention deficit-hyperactivity disorder: a voxel-based MRI study of regional grey and white matter volume. Psychiatry Res. 154, 171–180 10.1016/j.pscychresns.2006.09.006 17291727

[B93] McAlonan G. M. Cheung V. Cheung C. Suckling J. Lam G. Y. Tai K. S. (2005). Mapping the brain in autism: a voxel-based MRI study of volumetric differences and intercorrelations in autism. Brain 128, 268–276 10.1093/brain/awh332 15548557

[B94] McAlonan G. M. Suckling J. Wong N. Cheung V. Lienenkaemper N. Cheung C. (2008). Distinct patterns of grey matter abnormality in high-functioning autism and Asperger's syndrome. J. Child Psychol. Psychiatry 49, 1287–1295 10.1111/j.1469-7610.2008.01933.x 18673405

[B95] Minshew N. J. Keller T. A. (2010). The nature of brain dysfunction in autism: functional brain imaging studies. Curr. Opin. Neurol. 23, 124–130 10.1097/WCO.0b013e32833782d4 20154614 PMC2975255

[B96] Montes L. G. Ricardo-Garcell J. De la Torre L. B. Alcantara H. P. Garcia R. B. Acosta D. A. (2011). Cerebellar gray matter density in females with ADHD combined type: a cross-sectional voxel-based morphometry study. J. Atten. Disord. 15, 368–381 10.1177/1087054710366421 21490174

[B97] Mostofsky S. H. Reiss A. L. Lockhart P. Denckla M. B. (1998). Evaluation of cerebellar size in attention-deficit hyperactivity disorder. J. Child Neurol. 13, 434–439 10.1177/088307389801300904 9733289

[B98] Nickl-Jockschat T. Habel U. Michel T. M. Manning J. Laird A. R. Fox P. T. (2012). Brain structure anomalies in autism spectrum disorder–a meta-analysis of VBM studies using anatomic likelihood estimation. Hum. Brain Mapp. 33, 1470–1489 10.1002/hbm.21299 21692142 PMC4801488

[B99] Nicolson R. Fawcett A. Dean P. (2001). Developmental dyslexia: the cerebellar deficit hypothesis. Trends Neurosci. 24, 508–511 10.1016/S0166-2236(00)01896-8 11506881

[B100] Nicolson R. I. Fawcett A. J. (2007). Procedural learning difficulties: reuniting the developmental disorders? Trends Neurosci. 30, 135–141 10.1016/j.tins.2007.02.003 17328970

[B101] Nicolson R. I. Fawcett A. J. (2011). Dyslexia, dysgraphia, procedural learning and the cerebellum. Cortex 47, 117–127 10.1016/j.cortex.2009.08.016 19818437

[B102] Overmeyer S. Bullmore E. T. Suckling J. Simmons A. Williams S. C. Santosh P. J. (2001). Distributed grey and white matter deficits in hyperkinetic disorder: MRI evidence for anatomical abnormality in an attentional network. Psychol. Med. 31, 1425–1435 10.1017/S0033291701004706 11722157

[B103] Pernet C. R. Poline J. B. Demonet J. F. Rousselet G. A. (2009). Brain classification reveals the right cerebellum as the best biomarker of dyslexia. BMC Neurosci. 10:67 10.1186/1471-2202-10-67 19555471 PMC2713247

[B104] Rae C. Harasty J. A. Dzendrowskyj T. E. Talcott J. B. Simpson J. M. Blamire A. M. (2002). Cerebellar morphology in developmental dyslexia. Neuropsychologia 40, 1285–1292 10.1016/S0028-3932(01)00216-0 11931931

[B105] Ramnani N. (2006). The primate cortico-cerebellar system: anatomy and function. Nat. Rev. Neurosci. 7, 511–522 10.1038/nrn1953 16791141

[B106] Reiersen A. M. Constantino J. N. Volk H. E. Todd R. D. (2007). Autistic traits in a population-based ADHD twin sample. J. Child Psychol. Psychiatry 48, 464–472 10.1111/j.1469-7610.2006.01720.x 17501727

[B107] Richlan F. (2012). Developmental dyslexia: dysfunction of a left hemisphere reading network. Front. Hum. Neurosci. 6:120 10.3389/fnhum.2012.00120 22557962 PMC3340948

[B108] Richlan F. Kronbichler M. Wimmer H. (2009). Functional abnormalities in the dyslexic brain: a quantitative meta-analysis of neuroimaging studies. Hum. Brain Mapp. 30, 3299–3308 10.1002/hbm.20752 19288465 PMC2989182

[B109] Ritvo E. R. Freeman B. J. Scheibel A. B. Duong T. Robinson H. Guthrie D. (1986). Lower Purkinje cell counts in the cerebella of four autistic subjects: initial findings of the UCLA-NSAC Autopsy Research Report. Am. J. Psychiatry 143, 862–866 3717426 10.1176/ajp.143.7.862

[B110] Riva D. Annuziata S. Contarino V. Erbetta A. Aquino D. Bulgheroni S. (2013). Gray matter reduction in the vermis and CRUS-II is associated with social and interaction deficits in low-functioning children with autistic spectrum disorders: a VBM-DARTEL study. Cerebellum 12, 676–685 10.1007/s12311-013-0469-8 23572290

[B111] Riva D. Bulgheroni S. Aquino D. Di Salle F. Savoiardo M. Erbetta A. (2011). Basal forebrain involvement in low-functioning autistic children: a voxel-based morphometry study. AJNR Am. J. Neuroradiol. 32, 1430–1435 10.3174/ajnr.A2527 21700792 PMC7964346

[B112] Riva D. Giorgi C. (2000). The cerebellum contributes to higher functions during development: evidence from a series of children surgically treated for posterior fossa tumours. Brain 123, 1051–1061 10.1093/brain/123.5.1051 10775549

[B113] Rojas D. C. Peterson E. Winterrowd E. Reite M. L. Rogers S. J. Tregellas J. R. (2006). Regional gray matter volumetric changes in autism associated with social and repetitive behavior symptoms. BMC Psychiatry 6:56 10.1186/1471-244X-6-56 17166273 PMC1770914

[B114] Rommelse N. N. Franke B. Geurts H. M. Hartman C. A. Buitelaar J. K. (2010). Shared heritability of attention-deficit/hyperactivity disorder and autism spectrum disorder. Eur. Child Adolesc. Psychiatry 19, 281–295 10.1007/s00787-010-0092-x 20148275 PMC2839489

[B115] Rommelse N. N. J. Geurts H. M. Franke B. Buitelaar J. K. Hartman C. A. (2011). A review on cognitive and brain endophenotypes that may be common in autism spectrum disorder and attention-deficit/hyperactivity disorder and facilitate the search for pleiotropic genes. Neurosci. Biobehav. Rev. 35, 1363–1396 10.1016/j.neubiorev.2011.02.015 21382410

[B116] Rout U. K. Mungan N. K. Dhossche D. M. (2012). Presence of GAD65 autoantibodies in the serum of children with autism or ADHD. Eur. Child Adolesc. Psychiatry 21, 141–147 10.1007/s00787-012-0245-1 22323074

[B117] Salmond C. H. Ashburner J. Connelly A. Friston K. J. Gadian D. G. Vargha-Khadem F. (2005). The role of the medial temporal lobe in autistic spectrum disorders. Eur. J. Neurosci. 22, 764–772 10.1111/j.1460-9568.2005.04217.x 16101758

[B118] Salmond C. H. Vargha-Khadem F. Gadian D. G. de Haan M. Baldeweg T. (2007). Heterogeneity in the patterns of neural abnormality in autistic spectrum disorders: evidence from ERP and MRI. Cortex 43, 686–699 10.1016/S0010-9452(08)70498-2 17710821

[B119] Sasayama D. Hayashida A. Yamasue H. Harada Y. Kaneko T. Kasai K. (2010). Neuroanatomical correlates of attention-deficit-hyperactivity disorder accounting for comorbid oppositional defiant disorder and conduct disorder. Psychiatry Clin. Neurosci. 64, 394–402 10.1111/j.1440-1819.2010.02102.x 20546170

[B120] Schmitz N. Rubia K. Daly E. Smith A. Williams S. Murphy D. G. (2006). Neural correlates of executive function in autistic spectrum disorders. Biol. Psychiatry 59, 7–16 10.1016/j.biopsych.2005.06.007 16140278

[B121] Schweren L. J. de Zeeuw P. Durston S. (2013). MR imaging of the effects of methylphenidate on brain structure and function in Attention-Deficit/Hyperactivity Disorder. Eur. Neuropsychopharmacol. 23, 1151–1164 10.1016/j.euroneuro.2012.10.014 23165220

[B122] Seidman L. J. Biederman J. Liang L. Valera E. M. Monuteaux M. C. Brown A. (2011). Gray matter alterations in adults with attention-deficit/hyperactivity disorder identified by voxel based morphometry. Biol. Psychiatry 69, 857–866 10.1016/j.biopsych.2010.09.053 21183160 PMC3940267

[B123] Semrud-Clikeman M. Hooper S. R. Hynd G. W. Hern K. Presley R. Watson T. (1996). Prediction of group membership in developmental dyslexia, attention deficit hyperactivity disorder, and normal controls using brain morphometric analysis of magnetic resonance imaging. Arch. Clin. Neuropsychol. 11, 521–528 10.1093/arclin/11.6.521 14588457

[B124] Silani G. Frith U. Demonet J. Fazio F. Perani D. Price C. (2005). Brain abnormalities underlying altered activation in dyslexia: a voxel based morphometry study. Brain 128, 2453–2461 10.1093/brain/awh579 15975942

[B125] Simonoff E. Pickles A. Charman T. Chandler S. Loucas T. Baird G. (2008). Psychiatric disorders in children with autism spectrum disorders: prevalence, comorbidity, and associated factors in a population-derived sample. J. Am. Acad. Child Adolesc. Psychiatry 47, 921–929 10.1097/CHI.0b013e318179964f 18645422

[B126] Simos P. Breier J. Fletcher J. Foorman B. Bergman E. Fishbeck K. (2000). Brain activation profiles in dyslexic children during non-word reading: a magnetic source imaging study. Neurosci. Lett. 290, 61–65 10.1016/S0304-3940(00)01322-7 10925175

[B127] Siok W. T. Niu Z. Jin Z. Perfetti C. A. Tan L. H. (2008). A structural-functional basis for dyslexia in the cortex of Chinese readers. Proc. Natl. Acad. Sci. U.S.A. 105, 5561–5566 10.1073/pnas.0801750105 18391194 PMC2291101

[B128] Sivaswamy L. Kumar A. Rajan D. Behen M. Muzik O. Chugani D. (2010). A diffusion tensor imaging study of the cerebellar pathways in children with autism spectrum disorder. J. Child Neurol. 25, 1223–1231 10.1177/0883073809358765 20179000

[B129] Sonuga-Barke E. Bitsakou P. Thompson M. (2010). Beyond the dual pathway model: evidence for the dissociation of timing, inhibitory, and delay-related impairments in attention-deficit/hyperactivity disorder. J. Am. Acad. Child Adolesc. Psychiatry 49, 345–355 10.1016/j.jaac.2009.12.018 20410727

[B130] Steinbrink C. Vogt K. Kastrup A. Muller H. P. Juengling F. D. Kassubek J. (2008). The contribution of white and gray matter differences to developmental dyslexia: insights from DTI and VBM at 3.0 T. Neuropsychologia 46, 3170–3178 10.1016/j.neuropsychologia.2008.07.015 18692514

[B131] Stoodley C. J. MacMore J. Makris N. Sherman J. C. Schmahmann J. D. (2012a). Preliminary voxel-based lesion-symptom mapping in cerebellar stroke patients: Motor vs. cognitive outcomes, in Society for Neuroscience Annual Meeting (New Orleans, LA).

[B132] Stoodley C. J. Schmahmann J. D. (2009). Functional topography in the human cerebellum: a meta-analysis of neuroimaging studies. Neuroimage 44, 489–501 10.1016/j.neuroimage.2008.08.039 18835452

[B133] Stoodley C. J. Schmahmann J. D. (2010). Evidence for topographic organization in the cerebellum of motor control versus cognitive and affective processing. Cortex 46, 831–844 10.1016/j.cortex.2009.11.008 20152963 PMC2873095

[B134] Stoodley C. J. Stein J. F. (2011). The cerebellum and dyslexia. Cortex 47, 101–116 10.1016/j.cortex.2009.10.005 20060110

[B135] Stoodley C. J. Stein J. F. (2013). Cerebellar function in developmental dyslexia. Cerebellum 12, 267–276 10.1007/s12311-012-0407-1 22851215

[B136] Stoodley C. J. Valera E. M. Schmahmann J. D. (2012b). Functional topography of the cerebellum for motor and cognitive tasks: an fMRI study. Neuroimage 59, 1560–1570 10.1016/j.neuroimage.2011.08.065 21907811 PMC3230671

[B137] Strick P. L. Dum R. P. Fiez J. A. (2009). Cerebellum and nonmotor function. Annu. Rev. Neurosci. 32, 413–434 10.1146/annurev.neuro.31.060407.125606 19555291

[B138] Talairach J. Tournoux P. (1988). Co-Planar Stereotaxic Atlas of the Human Brain. 3-Dimensional Proportional System: An Approach to Cerebral Imaging. New York, NY: Thieme Medical Publishers, Inc

[B139] Tan G. C. Y. Doke T. F. Ashburner J. Wood N. W. Frackowiak R. S. J. (2010). Normal variation in fronto-occipital circuitry and cerebellar structure with an autism-associated polymorphism of CNTNAP2. Neuroimage 53, 1030–1042 10.1016/j.neuroimage.2010.02.018 20176116 PMC2941042

[B140] Tavano A. Grasso R. Gagliardi C. Triulzi F. Bresolin N. Fabbro F. (2007). Disorders of cognitive and affective development in cerebellar malformations. Brain 130, 2646–2660 10.1093/brain/awm201 17872929

[B141] Tomasi D. Volkow N. D. (2012). Abnormal functional connectivity in children with attention-deficit/hyperactivity disorder. Biol. Psychiatry 71, 443–450 10.1016/j.biopsych.2011.11.003 22153589 PMC3479644

[B142] Turkeltaub P. E. Eden G. F. Jones K. M. Zeffiro T. A. (2002). Meta-analysis of the functional neuroanatomy of single-word reading: method and validation. Neuroimage 16, 765–780 10.1006/nimg.2002.1131 12169260

[B143] Turkeltaub P. E. Eickhoff S. B. Laird A. R. Fox M. Wiener M. Fox P. (2012). Minimizing within-experiment and within-group effects in Activation Likelihood Estimation meta-analyses. Hum. Brain Mapp. 33, 1–13 10.1002/hbm.21186 21305667 PMC4791073

[B144] Valera E. Faraone S. Murray K. Seidman L. (2007). Meta-analysis of structural imaging findings in Attention-Deficit/Hyperactivity Disorder. Biol. Psychiatry 61, 1361–1369 10.1016/j.biopsych.2006.06.011 16950217

[B145] Valera E. M. Faraone S. V. Biederman J. Poldrack R. A. Seidman L. J. (2005). Functional neuroanatomy of working memory in adults with attention-deficit/hyperactivity disorder. Biol. Psychiatry 57, 439–447 10.1016/j.biopsych.2004.11.034 15737657

[B146] Valera E. M. Spencer R. M. Zeffiro T. A. Makris N. Spencer T. J. Faraone S. V. (2010). Neural substrates of impaired sensorimotor timing in adult attention-deficit/hyperactivity disorder. Biol. Psychiatry 68, 359–367 10.1016/j.biopsych.2010.05.012 20619827 PMC2917236

[B147] van Ewijk H. Heslenfeld D. J. Zwiers M. P. Buitelaar J. K. Oosterlaan J. (2013). Diffusion tensor imaging in attention deficit/hyperactivity disorder: a systematic review and meta-analysis. Neurosci. Biobehav. Rev. 36, 1093–1106 10.1016/j.neubiorev.2012.01.003 22305957

[B148] van Wingen G. A. van den Brink W. Veltman D. J. Schmaal L. Dom G. Booij J. (2013). Reduced striatal brain volumes in non-medicated adult ADHD patients with comorbid cocaine dependence. Drug Alcohol. Depend. 131, 198–203 10.1016/j.drugalcdep.2013.05.007 23726981

[B149] Verly M. Verhoeven J. Zink I. Mantini D. Peeters R. Deprez S. (2014). Altered functional connectivity of the language network in ASD: role of classical language areas and cerebellum. Neuroimage Clin. 4, 374–382 10.1016/j.nicl.2014.01.008 24567909 PMC3930113

[B150] Vidyasagar T. R. Pammer K. (2010). Dyslexia: a deficit in visuo-spatial attention, not in phonological processing. Trends Cogn. Sci. 14, 57–63 10.1016/j.tics.2009.12.003 20080053

[B151] Vinckenbosch E. Robichon F. Eliez S. (2005). Gray matter alteration in dyslexia: converging evidence from volumetric and voxel-by-voxel MRI analyses. Neuropsychologia 43, 324–331 10.1016/j.neuropsychologia.2004.06.023 15707610

[B152] Vloet T. D. Gilsbach S. Neufang S. Fink G. R. Herpertz-Dahlmann B. Konrad K. (2010). Neural mechanisms of interference control and time discrimination in attention-deficit/hyperactivity disorder. J. Am. Acad. Child Adolesc. Psychiatry 49, 356–367 10.1016/j.jaac.2010.01.004 20410728

[B153] Vollm B. A. Taylor A. N. Richardson P. Corcoran R. Stirling J. McKie S. (2006). Neuronal correlates of theory of mind and empathy: a functional magnetic resonance imaging study in a nonverbal task. Neuroimage 29, 90–98 10.1016/j.neuroimage.2005.07.022 16122944

[B154] Vossel S. Geng J. J. Fink G. R. (2014). Dorsal and ventral attention systems: distinct neural circuits but collaborative roles. Neuroscientist 20, 150–159 10.1177/1073858413494269 23835449 PMC4107817

[B155] Waiter G. D. Williams J. H. Murray A. D. Gilchrist A. Perrett D. I. Whiten A. (2004). A voxel-based investigation of brain structure in male adolescents with autistic spectrum disorder. Neuroimage 22, 619–625 10.1016/j.neuroimage.2004.02.029 15193590

[B156] Willcutt E. G. Betjemann R. S. McGrath L. M. Chhabildas N. A. Olson R. K. DeFries J. C. (2010). Etiology and neuropsychology of comorbidity between RD and ADHD: the case for multiple-deficit models. Cortex 46, 1345–1361 10.1016/j.cortex.2010.06.009 20828676 PMC2993430

[B157] Wilson L. B. Tregellas J. R. Hagerman R. J. Rogers S. J. Rojas D. C. (2009). A voxel-based morphometry comparison of regional gray matter between fragile X syndrome and autism. Psychiatry Res. 174, 138–145 10.1016/j.pscychresns.2009.04.013 19853418 PMC2783567

[B158] Wolf R. C. Plichta M. M. Sambataro F. Fallgatter A. J. Jacob C. Lesch K. P. (2009). Regional brain activation changes and abnormal functional connectivity of the ventrolateral prefrontal cortex during working memory processing in adults with attention-deficit/hyperactivity disorder. Hum. Brain Mapp. 30, 2252–2266 10.1002/hbm.20665 19107748 PMC6870879

[B159] Yang P. Wang P. N. Chuang K. H. Jong Y. J. Chao T. C. Wu M. T. (2008). Absence of gender effect on children with attention-deficit/hyperactivity disorder as assessed by optimized voxel-based morphometry. Psychiatry Res. 164, 245–253 10.1016/j.pscychresns.2007.12.013 19013775

[B160] Yeo B. T. Krienen F. M. Sepulcre J. Sabuncu M. R. Lashkari D. Hollinshead M. (2011). The organization of the human cerebral cortex estimated by intrinsic functional connectivity. J. Neurophysiol. 106, 1125–1165 10.1152/jn.00338.2011 21653723 PMC3174820

[B161] Yu K. K. Cheung C. Chua S. E. McAlonan G. M. (2011). Can Asperger syndrome be distinguished from autism? An anatomic likelihood meta-analysis of MRI studies. J. Psychiatry Neurosci. 36, 412–421 10.1503/jpn.100138 21406158 PMC3201995

[B162] Yun H. S. Park M. S. Ji E. S. Kim T. W. Ko I. G. Kim H. B. (2014). Treadmill exercise ameliorates symptoms of attention deficit/hyperactivity disorder through reducing Purkinje cell loss and astrocytic reaction in spontaneous hypertensive rats. J. Exerc. Rehabil. 28, 22–30 10.12965/jer.140092 24678501 PMC3952832

[B163] Zatorre R. J. Fields R. D. Johansen-Berg H. (2012). Plasticity in gray and white: neuroimaging changes in brain structure during learning. Nat. Neurosci. 15, 528–536 10.1038/nn.3045 22426254 PMC3660656

