# Supplementary material for: Distinct regions of the cerebellum show gray matter decreases in autism, ADHD, and developmental dyslexia
Source: Front Syst Neurosci. 2014 May 20;8:92. doi: 10.3389/fnsys.2014.00092 (PMC4033133; doi:10.3389/fnsys.2014.00092)
Supplement: Supplementary file 1 [file DataSheet1.PDF]

Supplementary Table 1. Whole-brain ALE results in ASD. ALE maps were thresholded at $p<0.001$ with a minimum cluster size of 50 voxels. x, y, z = MNI coordinates.

ASD<TD

Cluster #	Volume (mm^3)	ALE value x 10^{-3}	x	y	z	Estimated anatomical location (Talairach Daemon)
1	664	15.61	0	-66	40	Left cuneus, Brodmann area 7
2	368	11.50	-24	-44	-52	Left Cerebellum VIIIB
3	352	13.98	54	-54	-36	Right Cerebellum Crus I
4	328	12.85	-2	-60	-40	Midline Cerebellum IX
5	224	11.05	28	-24	70	Right Postcentral Gyrus, Brodmann area 3
6	216	10.75	-56	-28	18	Left Postcentral Gyrus, Brodmann area 40
7	200	10.47	0	4	-6	Left Anterior Cingulate, Brodmann area 25
8	120	9.62	-30	52	14	Left Middle Frontal Gyrus, Brodmann area 10
9	120	10.97	0	-68	16	Left Posterior Cingulate, Brodmann area 30
10	104	9.40	30	-10	-16	Right Amygdala
11	96	10.41	40	-85	22	Right Middle Occipital Gyrus, Brodmann area 19
12	88	10.97	6	-72	12	Right Cuneus, Brodmann area 30
13	56	10.41	30	-84	-24	Right Cerebellum Crus I
14	56	10.41	28	-84	-18	Right Cerebellum Crus I
15	56	10.41	52	-62	-16	Right Fusiform Gyrus, Brodmann area 37
16	56	10.41	-34	-94	-10	Left Inferior Occipital Gyrus, Brodmann area 18
17	56	10.41	18	-92	-10	Right Lingual Gyrus, Brodmann area 18
18	56	8.95	16	2	-2	Right Medial Globus Pallidus
19	56	10.41	12	-96	2	Right Lingual Gyrus, Brodmann area 17
20	56	9.38	2	44	38	Left Medial Frontal Gyrus, Brodmann area 8

ASD>TD

Cluster #	Volume (mm^3)	ALE value x 10^{-3}	x	y	z	Estimated anatomical location (Talairach Daemon)
1	768	16.08	-44	4	-32	Left Middle Temporal Gyrus, Brodmann area 21
2	328	11.56	-20	18	-6	Left Putamen
		11.26	-10	16	-10	Left Caudate Head
3	296	13.93	16	-48	20	Right Posterior Cingulate, Brodmann area 29
4	288	14.73	-24	-26	8	Left Thalamus, Pulvinar
5	224	13.15	40	-20	-8	Right Claustrum
6	208	12.52	-16	32	44	Left Superior Frontal Gyrus, Brodmann area 8
7	200	11.91	26	50	16	Right Superior Frontal Gyrus, Brodmann area 10
8	184	12.36	26	-90	-8	Right Middle Occipital Gyrus, Brodmann area 18
9	176	11.92	-22	-92	6	Left Lingual Gyrus, Brodmann area 17
10	120	11.15	50	-2	42	Right Precentral Gyrus, Brodmann area 6
11	56	10.52	-58	-22	-16	Left Middle Temporal Gyrus, Brodmann area 21
12	56	10.55	-34	32	-2	Left Inferior Frontal Gyrus, Brodmann area 47

13	56	10.63	-38	-18	50	Left Postcentral Gyrus, Brodmann area 3

Supplementary Table 2. Whole-brain ALE results in ADHD. ALE maps were thresholded at p<0.001 with a minimum cluster size of 50 voxels. x, y, z = MNI coordinates.

ADHD<TD

Cluster #	Volume (mm^3)	ALE value x 10^{-3}	x	y	z	Estimated anatomical location (Talairach Daemon)
1	464	10.75	28	-2	4	Right Putamen
2	232	10.11	10	30	-20	Right Medial Frontal Gyrus Brodmann area 11
3	208	10.28	-22	-4	-26	Left Amygdala
4	88	9.93	10	12	8	Right Caudate Body
5	88	9.91	-6	-98	14	Left Cuneus Brodmann area 18
6	80	9.67	-16	-48	-45	Left Cerebellum lobule IX
7	80	9.67	21	-86	2	Right Lingual Gyrus, Brodmann area 17
8	80	9.82	-8	18	8	Left Caudate Body
9	80	8.56	-26	-23	68	Left Precentral Gyrus, Brodmann area 4
		8.01	-25	-18	69	Left Precentral Gyrus, Brodmann area 4
10	64	8.99	18	-48	-46	Right Cerebellum lobule IX
11	64	8.01	-33	-27	-29	Left Parahippocampal Gyrus, Brodmann area 36
12	64	8.05	18	28	-18	Right Inferior Frontal Gyrus, Brodmann area 47
13	64	8.01	-19	-37	63	Left Paracentral Lobule, Brodmann area 5

ADHD>TD

Cluster #	Volume (mm^3)	ALE value x 10^{-3}	x	y	z	Estimated anatomical location (Talairach Daemon)
1	288	7.11	-2	-15	5	Left Thalamus, Medial Dorsal Nucleus
2	288	7.27	-13	-27	40	Left Cingulate Gyrus, Brodmann area 31
3	264	7.73	-14	-54	46	Left Precuneus, Brodmann area 7
4	264	7.73	-34	-34	48	Left Inferior Parietal Lobule, Brodmann area 40
5	256	7.05	-49	-21	23	Left Insula, Brodmann area 13
6	256	7.05	-15	-45	37	Left Precuneus, Brodmann area 31
7	256	6.90	45	-15	37	Right Precentral Gyrus, Brodmann area 4
8	224	7.50	-14	-39	60	Left Precuneus, Brodmann area 7

Supplementary Table 3. Whole-brain ALE results in developmental dyslexia. ALE maps were thresholded at p<0.001 with a minimum cluster size of 50 voxels. x, y, z = MNI coordinates.

**Dyslexia< TD** Cluster #	Volume (mm^3)	ALE value x 10^{-3}	x	y	z	Estimated anatomical location (Talairach Daemon)
1	392	10.18	-26	-50	-32	Left Cerebellum lobule VI
2	160	8.87	-34	42	-20	Left Middle Frontal Gyrus, Brodmann area 11
3	160	8.98	-56	-52	2	Left Middle Temporal Gyrus, Brodmann area 22
4	136	8.61	64	-34	18	Right Insula, Brodmann area 13
5	80	7.65	-54	4	-16	Left Middle Temporal Gyrus, Brodmann area 21
6	80	7.65	-32	18	10	Left Insula, Brodmann area 13
7	80	7.96	-42	0	18	Left Insula, Brodmann area 13
8	80	7.65	-32	31	28	Left Middle Frontal Gyrus, Brodmann area 9
9	72	8.12	-68	-26	34	Left Inferior Parietal Lobule, Brodmann area 40
10	64	6.73	32	-52	-20	Right Cerebellum lobule VI
11	64	7.09	2	-102	0	Left Lingual Gyrus, Brodmann area 18
12	64	7.77	46	14	12	Right Insula, Brodmann area 13
13	64	7.90	-22	4	18	Left Putamen
14	64	6.73	-55	-51	49	Left Inferior Parietal Lobule, Brodmann area 40

**Dyslexia>TD** Cluster #	Volume (mm^3)	ALE value x 10^{-3}	x	y	z	Estimated anatomical location (Talairach Daemon)
1	352	8.97	-60	-60	5	Left Middle Temporal Gyrus, Brodmann area 21
2	216	7.36	-6	50	18	Left Medial Frontal Gyrus, Brodmann area 9
3	216	7.36	14	-46	44	Right Cingulate Gyrus, Brodmann area 31
4	216	7.36	6	12	54	Right Superior Frontal Gyrus, Brodmann area 6
5	160	7.15	-50	-26	3	Left Superior Temporal Gyrus
6	160	6.94	12	51	5	Right Medial Frontal Gyrus, Brodmann area 10
7	160	7.15	56	0	23	Right Precentral Gyrus, Brodmann area 6
8	160	6.94	20	11	51	Right Medial Frontal Gyrus, Brodmann area 6
9	160	7.15	18	-38	60	Right Paracentral Lobule, Brodmann area 5
10	136	6.48	18	-24	78	Right Precentral Gyrus, Brodmann area 4
